# Single-shot digital optical fluorescence phase conjugation through forward multiple-scattering samples

**DOI:** 10.1126/sciadv.adi1120

**Published:** 2024-01-19

**Authors:** Tengfei Wu, Yixuan Zhang, Baptiste Blochet, Payvand Arjmand, Pascal Berto, Marc Guillon

**Affiliations:** ^1^ Saints-Pères Paris Institute for the Neurosciences, CNRS UMR 8003, Université Paris Cité, 45 rue des Saints-Pères, Paris 75006, France.; ^2^ Sorbonne Université, CNRS, INSERM, Institut de la Vision, 17 Rue Moreau, Paris 75012, France.; ^3^ Institut Universitaire de France (IUF), Paris 75007, France.

## Abstract

Aberrations and multiple scattering in biological tissues critically distort light beams into highly complex speckle patterns. In this regard, digital optical phase conjugation (DOPC) is a promising technique enabling in-depth focusing. However, DOPC becomes challenging when using fluorescent guide stars for four main reasons: the low photon budget available, the large spectral bandwidth of the fluorescent signal, the Stokes shift between the emission and the excitation wavelength, and the absence of reference beam preventing holographic measurement. Here, we demonstrate the possibility to focus a laser beam through multiple-scattering samples by measuring speckle fields in a single acquisition step with a reference-free, high-resolution wavefront sensor. By taking advantage of the large spectral bandwidth of forward multiply scattering samples, digital fluorescence phase conjugation is achieved to focus a laser beam at the excitation wavelength while measuring the broadband speckle field arising from a micrometer-sized fluorescent bead.

## Teaser

Multispectral speckle and laser beams are measured and focused through multiple-scattering samples using a wavefront sensor.

## INTRODUCTION

Laser light focusing inside or through aberrant and scattering sample is a major challenge which is of interest for in-depth imaging (*1*), photostimulation (*2*), or phototherapy (*3*). At depth larger than the scattering mean free path ℓ*_s_* [typically ≃100 μm in brain tissues (*4*)], light is scrambled into a complex random-like speckle pattern. Imaging ability is thus critically altered by reduced signal-to-noise ratios, even under nonlinear photoexcitation (*5*).

In-depth light control has then been addressed both by adaptive optics (AO) (*6*) and wavefront shaping (*7*) approaches. AO efficiently compensates for optical aberrations. It relies on the weak turbulence assumption and considers that a spatially coherent light beam (e.g., originating from a guide star or a laser) only experiences smooth wavefront distortions. This assumption remains valid shallow in the sample (*z* ≲ ℓ*_s_*), in transverse planes conjugated with smooth and large-scaled refractive index mismatches. For discontinuous, small-scaled or typical volumetric refractive index mismatches, multiple light path trajectories (i.e., scattering) are involved and interfere, resulting not only in wavefront distortions but also in highly contrasted intensity fluctuations. At depths larger than ℓ*_s_*, the fraction of scattered light exceeds ballistic energy, potentially by several orders of magnitude. More complex optical control is then demanded, referred as “wavefront shaping” techniques in the literature (*7*). A system compensating tissue distortion at various depths must be able to measure and correct both low varying wavefront distortions—attributed to aberrations—and highly scrambled wavefronts induced by scattering.

Aberrations and scattering corrections rely on the principle of phase conjugation. The latter is based on the use of a spatial light modulator (SLM) placed in the excitation and/or the detection path of an imaging system. Phase conjugation can then be achieved either iteratively or by direct wavefront measurement. In the iterative approaches, the correcting pattern is either obtained by fully characterizing the transmission matrix from several measurements (*8*, *9*) or by sequentially updating the pattern at the SLM to maximize a proper metric that ensures focusing. This metric can be the intensity at a given position (*10*), a nonlinear signal (*11*–*14*), an image quality metric (*15*, *16*), or a variance contrast (*17*). Those approaches have proven successful to correct both scattering and aberration in a wide range of scenarios (*7*): coherent (*10*), incoherent (*18*), with (*18*), or without guide stars (*11*, *19*). However, these techniques demand multiple sequential measurements at the expense of speed, which ultimately becomes an issue, especially in the case of rapidly evolving media such as living animals (*20*).

Alternatively, direct wavefront conjugation is also possible and potentially faster. It consists in first measuring the wavefront arising from a guide star (and emerging from the scattering medium) and then to correct wavefront distortions in a second step. This solution is effective when using coherent guide stars (*21*, *22*) since interferometric measurements is then possible. Conversely, for incoherent guide stars such as fluorescent, the outgoing wave field can only be measured with reference-less wavefront sensing techniques. However, in this case, only multi-acquisition schemes combined with phase-retrieval optimization algorithms have allowed measuring complex fluorescence wavefronts through scattering media so far (*23*, *24*), and single-shot fluorescence wave field measurement has been limited to low-order aberration compensation in AO schemes (*6*, *25*, *26*). Noteworthy, direct scattering compensation by single-shot amplitude-only acquisition was also demonstrated on the basis of a common-path speckle-interferometric measurement (*27*) but then ignoring ballistic low-order aberrated light. Furthermore, in (*27*), digital optical phase conjugation (DOPC) by binary amplitude-only beam modulation was achieved, at the expense of a drastic loss in focusing efficiency (*28*, *29*). Therefore, a single-shot measurement of both aberrant and scattered fluorescence wavefronts originating from an incoherent guide star hidden by a complex medium has never been achieved. The solution we propose relies on a typical AO scheme.

Fluorescence has established itself as the gold standard optical contrast for biomicroscopy since being minimally invasive, biospecific, compatible with living samples, and even having the possibility to be genetically encoded through an increasing number of optogenetic tools, including a large choice of fluorescent proteins with a wide range of photosensitive properties. For this reason, fluorescence has also been primarily exploited to perform AO and compensate low-order aberrations (*6*). In bioimaging, AO is typically implemented by measuring the wavefronts with a Shack-Hartman wavefront sensor (WFS) (*30*, *31*). WFS are compact single-shot reference-free instruments, compatible with broadband light sources and providing the complex amplitude of a light beam (phase and intensity). They are thus ideally suited to measure fluorescence signals. WFS actually measures the wavefront gradient and thus requires a numerical integration step. So far, the use of WFS has mostly been limited to smooth and low-order aberrations measurements (*6*, *25*, *32*), although they have proved to be capable of providing high spatial resolution of smooth objects (*33*, *34*). In the context of astronomy, it was shown that in the case of strong turbulences, singular points of zero intensity associated with screw-phase dislocations (or optical vortices) appear and degrade AO performances (*35*), especially because deformable mirrors cannot compensate phase dislocations. AO, in its traditional implementation, has thus only been considered for compensating the first few low-order Zernike polynomials contribution to aberrations (*25*) and has long ignored the contribution of optical vortices to the phase structures, so preventing its use for compensating high-order multiple-scattering processes. Measuring random speckle wave fields has long remained a challenge because of the high spatial density of intrinsic optical vortices (*36*–*38*), which are responsible for a so-called hidden phase (*39*) canceled by the integration step (*40*). Optical vortices are associated with a vanishing intensity at the singularity location and a nonconservative and diverging grid-distortion vector field. Numerical (*41*) and experimental (*42*) efforts have been made to detect optical vortices using Shack-Hartmann WFS, but despite Fried’s suggestion to reconstruct the corresponding “hidden-phase” (*39*), only individual isolated vortices could be successfully restored in practical experiments (*43*–*45*). Recently, we proposed a solution that experimentally demonstrated the possibility to quantitatively rebuild complex wave fields containing high densities of optical vortices (*46*). By performing image processing of the Helmholz decomposition and relying on the quantization of optical vortex charges, we demonstrated accurate experimental speckle measurement with a reference-less WFS (see fig. S3).

Although WFS are compatible with broadband guide stars, performing high-resolution DOPC using broadband light beams like fluorescent signals and/or ultrashort laser pulses through multiple-scattering samples is complicated. Multiple scattering usually involves large distributions of path length trajectories and thus results in narrow spectral correlation bandwidths, and blurred broadband speckle patterns of low contrast whose phase and amplitude are not defined. Nevertheless, biological tissues are multiple-scattering samples having the specific property to exhibit large anisotropy factors (*4*). They can be typically modeled numerically by stacking thin low-angle–scattering phase plates (*47*–*49*). We have demonstrated that despite the multiple-scattering process, the large anisotropy factor *g* of biological tissues is responsible for large spectral bandwidths (*49*–*51*) scaling as Δλ = λ^2^ℓ^∗^/*L*^2^, where *L* is the slab thickness (ℓs ≪ *L* ≪ ℓ^∗^) and ℓ^∗^ = ℓs/(1 − *g*) is the transport mean-free path. An “achromatic” speckle plane lying in a virtual image plane located at two-thirds of the slab thickness was identified (see fig. S12) (*49*, *51*). The intermediate regime of biological tissues thus bridges the gap between the community of AO and wavefront shaping (*12*, *52*–*55*) by involving both the contribution of ballistic light (altered by geometrical aberrations) on the one hand and the contribution of multiply scattered light on the other hand.

Here, we demonstrate the possibility to compensate multiple scattering in a single-shot DOPC experiment in the absence of a reference beam. The system we use, based on a high-resolution WFS, is shown to be compatible with broadband light sources like fluorescent guide stars. This demonstration is based on a rigorous quantitative analysis of spectral correlations of multiply forward scattering samples and the resulting highly multimodal speckle beams. First, we demonstrate the key contribution of optical vortices to the wave field structure for phase conjugation applications. Second, we discuss the robustness of speckle phase conjugation to spectral shift thanks to the large spectral correlation width of forward multiply scattering samples (*49*–*51*). Third, DOPC performances are experimentally investigated as a function of the required number of photons per spatial modes. Last, on the basis of the former quantitative analysis, we demonstrate digital (30-nm-wide) fluorescence phase conjugation (DFPC) through 500 μm of paraffin samples.

## RESULTS

### DOPC with a WFS

**Fig. 1.**
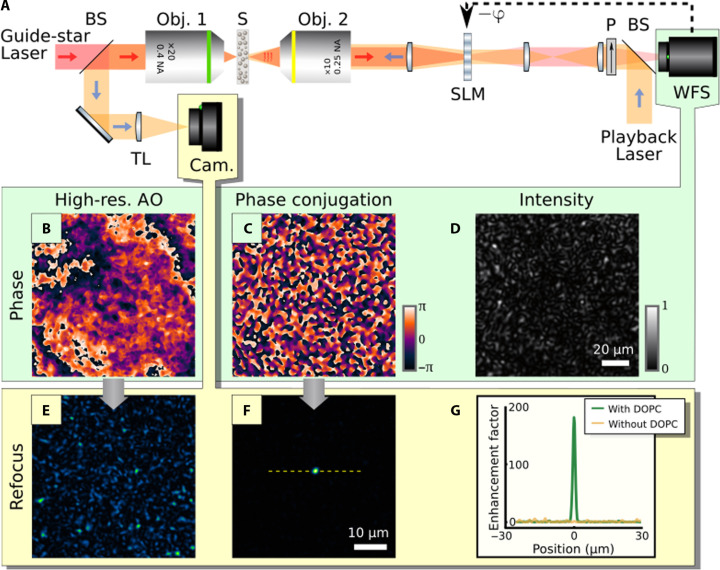
Principle of DOPC with a WFS. Scheme of the DOPC experiment (**A**). A guide star laser beam is focused by an objective lens (Obj. 1) onto the input face of a scattering sample S. The scattered light, collected by a second objective (Obj. 2), is sent onto a phase-only SLM conjugated to a WFS through a polarizer P. The phase (**C**) and the intensity (**D**) of the beam are measured while the SLM is exhibiting a flat phase. The smooth contribution of phase (C) is also rebuilt (**B**) for comparison. In the second step, a playback laser beam is injected backward thanks to a pellicle beam splitter. With smooth phase modulation at the SLM, a speckle pattern (**E**) is observed on the camera (Cam.). When displaying the measured phase pattern (C) onto the SLM, a sharp focus is observed behind the 720-μm-thick spinal cord slice from a mouse (**F**), exhibiting a ≃200 enhancement factor (**G**). BS, beam splitter. TL, tube lens.

The scheme of the experiment is shown in Fig. 1. A DOPC microscope was built up on the basis of a liquid-crystal SLM (LCoS X10468-01, Hamamatsu, Japan) conjugated with a high-resolution WFS in an “in-line” configuration to simplify the alignment of the WFS and SLM (see fig. S7) (*56*, *57*). In contrast with usual “pupil” AO systems, in “conjugated AO,” both the SLM and the WFS are conjugated to the scattering sample plane (*56*). As a result, the maximum number of spatial modes arriving at the WFS does not depend on the sample but is solely limited by the pupil size of the imaging objective lens (obj. 1 in Fig. 1). For usual pupil AO designs, the speckle grain size at the SLM/WFS decreases with an increasing size of the illuminated sample region, resulting in correspondingly large number of modes. This chosen “conjugate” AO configuration further allows us to conjugate both the WFS and the SLM to the achromatic plane of the forward scattering samples, which also turns out to be the best conjugating plane for optimizing the isoplanatic patch (*58*).

The WFS we use here is a homemade quadri-wave lateral shearing interferometer (*33*) made of a checkerboard phase grating conjugated to a plane located at a few millimeters from a 4Mpx sCMOS camera (Prime BSI Express, Teledyne-Photometrics). The possibility to accurately measure the full speckle phase pattern from a WFS based on the algorithm of (*46*) is shown in fig. S3 by comparing with a digital holographic measurement. In the DOPC setup, the maximum number of spatial modes arriving at the WFS was tuned to ≃3000 by optical design. This value was chosen on the basis of a prior quantitative experimental characterization of the WFS using the setup shown in fig. S1. Also, on the basis of the experimental results of this first scaling step, the expected evolution of the DOPC performances is plotted as a function of the number of modes in fig. S4. The process for optimizing the distance between the phase mask grating and the camera is described in fig. S6.

First, we demonstrate DOPC with a WFS at a single monochromatic wavelength. In Fig. 1, the principle is illustrated using a fixed 720-μm-thick spinal cord slice from a mouse as a sample. In a first step, a guide star point source is created at the rear of the tissue by focusing a 635-nm diode laser beam, after prior spatial cleaning through a monomode fiber (see fig. S5). A flat phase is displayed onto the SLM to record the output wave field with the WFS, intensity (Fig. 1D) and phase (Fig. 1C). In a second step, the phase measured at the WFS (Fig. 1C) is conjugated and addressed to the SLM, which is then illuminated by a back-propagating playback laser at the same wavelength. A sharp focus is then observed at the readout camera (Fig. 1F) exhibiting an enhancement factor (*10*, *13*) as large as *I*_focus_/ < *I*_speckle_ > ≃ 200 (Fig. 1G). This value can be compared to the maximum theoretical expectation that is qualitatively given by the number of measured and controlled modes (*10*, *59*). In our system, this number was set to ≃3000 by optical design (a factor ≃15 above the experimental result and a factor ≃10 above one might have been expecting from prior calibration experiment; fig. S4). The observed difference between the experimental enhancement factor and its theoretical maximum value may involve partial depolarization of the playback laser by the scattering medium, slight misalignment between the SLM and the WFS (fig. S7), and imperfect wavefront reconstruction. When ignoring the contribution of optical vortices in the phase reconstruction process (Fig. 1B), such as currently done by all Shack-Hartmann–based AO systems, no focus is obtained (Fig. 1E). The reason is that the fraction of ballistic light through this sample is negligible. This result demonstrates that high-resolution WFS enables reference-less single-shot DOPC through multiply scattering media. The ability to compensate multiple scattering is substantiated by the size of the isoplanatic patch, measured to be as small as a single speckle grain size (fig. S9). DOPC through a multimode fiber, of interest for microendoscopy techniques, is also demonstrated in fig. S10. The dimensions of the focal spot in Fig. 1F were measured to be 1.2 μm full width at half maximum (FWHM) and 1.5 μm FWHM along two orthogonal directions, respectively. These values are very close to the diffraction limit achievable with our objective lens (Obj. 2 in Fig. 1A): 0.51λ/NA = 1.31 μm, where NA is the numerical aperture of Obj. 2. The possibility to get focal spot dimensions smaller than the NA of the focusing lens is made possible thanks to the scattering medium increasing the effective focusing aperture (*10*). In fig. S8, we further demonstrate that using computer-generated holography, it is possible to exploit the angular memory effect of a diffuser to project more complex intensity patterns through the scattering sample.

### Contribution of optical vortices to DOPC

**Fig. 2.**
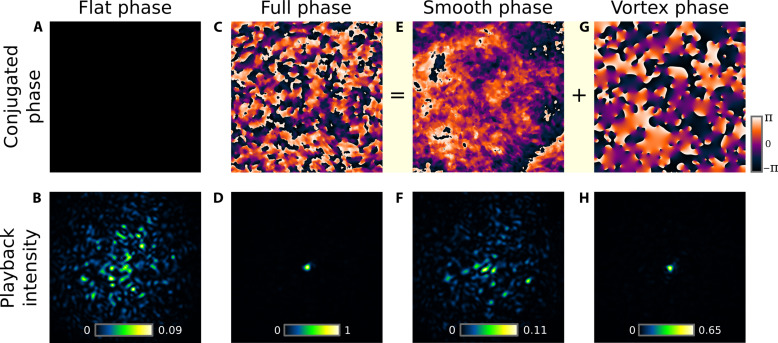
Importance of the optical vortices for efficient DOPC through an out-of-focus 1° surface diffuser. The SLM and WFS are conjugated to the virtual image of the guide star focus. Displaying a flat phase on the SLM (**A**) results in a speckle at the playback camera (**B**), while full phase compensation (**C**) yields a focus (**D**) containing 25% of light energy (amplitude set to one unit). The smooth contribution to the phase (**E**), typically rebuilt in AO experiments, does not provide a focus (**F**), while the vortex contribution to the phase (**G**) does (**H**).

With a Shack-Hartmann–like WFS, the wavefront is obtained by integrating the measured gradient vector field. Usual reconstruction algorithms only considered the curl-free contribution of the vector field, resulting in a conservative potential (*40*, *60*). The nonconservative solenoidal contribution of optical vortices, responsible for the so-called hidden phase (*39*), has so far been ignored in typical AO schemes. Here, we thus specifically study the contribution of both the conservative phase and the hidden-phase to DOPC performances. Results are shown in Fig. 2. A speckle is created by illuminating a holographic diffuser (1° diffuser, Edmund Optics) with a collimated laser beam. The diffuser was defocused by 2 mm from the conjugate plane of the SLM/WFS, yielding a speckle pattern containing many optical vortices. When a flat phase is displayed onto the SLM, the playback laser beam yields a fully developed speckle pattern on the camera (Fig. 2, A and B). When conjugating the full phase measured at the WFS including both phase contributions, a focus is obtained containing 25% of light energy (Fig. 2, C and D). Its amplitude was normalized for comparison with other cases. When considering only the smooth (i.e., conservative) contribution of the phase, which is solely rebuilt in AO systems, the laser energy is slightly concentrated to the central region, but no focus is obtained in this scattering condition that cannot be considered as low-order optical abberations (Fig. 2, E and F). In contrast, we observed that the vortex phase allows focusing ≃2/3 of the maximum energy, corroborating the idea that the structure of phase in speckle patterns is driven by vortex phase singularities (*61*). Experimentally, the fraction of focused energy was calculated by computing the ratio between the energy in the focused spot and the integration of the whole transmitted speckle signal obtained without phase conjugation, which fully falls in the field of view of our camera for forward scattering media (see fig. S15). Hereafter, we characterize DOPC performances by measuring the fraction of refocused energy rather than the enhancement factor. The enhancement factor is more suitable to cases where the number of controlled modes is much smaller than the number of modes of the scattering medium, which is the case of the sample used in Fig. 1. Conversely, for more forward scattering samples, the total transmitted energy is measurable, and the fraction of refocused energy becomes comparable to one. This result demonstrates that rebuilding the nonconservative contribution of the gradient vector field measured by WFS is of critical importance to efficiently compensate scattering in DOPC experiments.

### DOPC from a spectrally detuned guide star

In constrast to monochromatic DOPC that we have just demonstrated, the implementation of DOPC with a fluorescent guide star faces the specific challenge of robustness to spectral width and detuning. It is indeed necessary to be able to both measure broadband speckles and refocus a laser beam at a wavelength shorter than the average fluorescence wavelength to take into account the ≃25 nm Stokes’ shift of typical organic fluorophores. Then, we considered studying the robustness of DOPC to spectral detuning by shifting the wavelength of the playback laser as compared to the guide stars. In a former article, we showed that a fixed 1-mm-thick brain slice can exhibit a spectral correlation width as large as ≃200 nm (*50*). Although experiencing multiple-scattering events over optical trajectories much larger than the wavelength, we identified that, in virtue of the large anisotropy factor of such media (*g* close to 1), all trajectories are snake-like (*62*), and the dispersion in trajectories path lengths is much smaller than their average length (*51*). Consequently, the spectral bandwidth, scaling as the inverse of the dispersion in optical path delays, was derived to be $\Delta \lambda =\frac{\lambda^{2}\ell^{*}}{L^{2}}$ . Both analytical derivation (*51*) and numerical simulations (*49*), supported by experimental results, demonstrated that the speckles generated through the scattering sample had a spectral correlation maximum in a virtual plane located at two-thirds of the slab thickness. Noteworthy, not only speckle intensities are achromatic in this achromatic plane but also their spatial phases (*51*) and even their spectral phases (*49*).

**Fig. 3.**
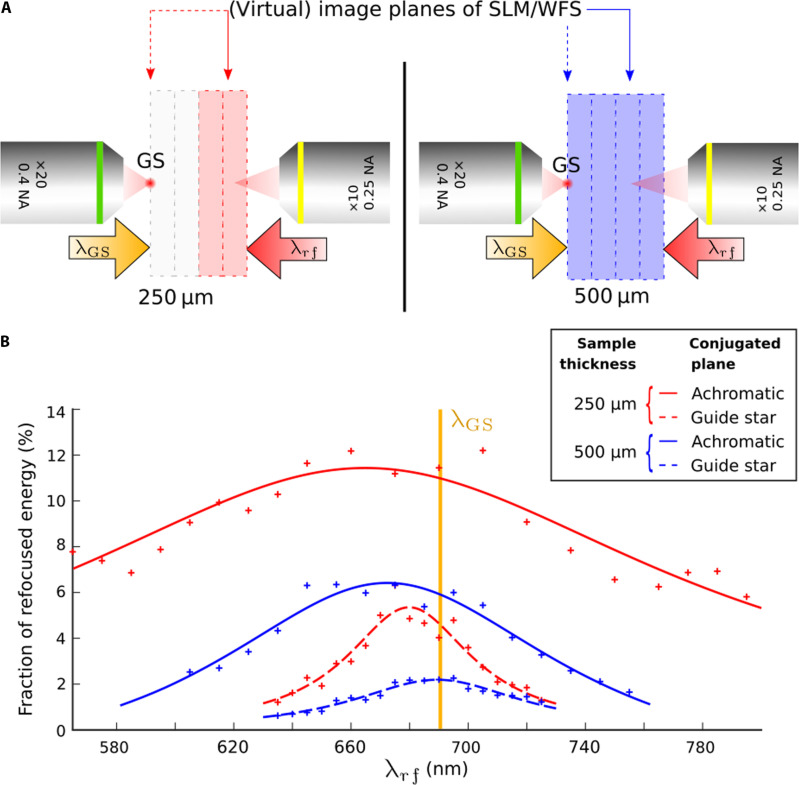
Fraction of refocused energy as a function of spectral detuning between the refocusing laser λ_*rf*_ and the guide star laser (λ_*GS*_ = 690/5nm). **Samples consist in 250-μm-thick (red curves) and 500-μm-thick (blue curves) samples of paraffin. In both cases, refocusing efficiency is plotted in two conditions: with the SLM/WFS conjugated to the achromatic plane [solid arrows in (**A**) and solid fitting lines in (**B**)] and with the guide star plane [dashed arrows in (A) and dashed fitting lines in (B)].

We thus conjugated our SLM/WFS to this achromatic plane to both maximize coherence of the broadband guide star speckle and to make DOPC robust to spectral detuning. To demonstrate the relevance of this configuration, we compare its performances to the case where the SLM/WFS are conjugated to the guide star plane. Data shown in Fig. 3 are obtained for two sample thicknesses, *L* = 250 μm and *L* = 500 μm, obtained by stacking two and four layers of parafilm, respectively. The achromatic plane location, which we evidenced experimentally (see fig. S12), is identified by solid line arrows in Fig. 3A and the guide star planes by dashed line arrows. A focused laser spot having a fixed wavelength λ_GS_ = 690 nm was used as a guide star, and DOPC performances measured as the playback laser were spectrally scanned (λ*_rf_*). Experimentally measured fraction of refocused energy are plotted in Fig. 3B and fitted by Lorentzian profiles [in agreement with theory (*51*)]

$$F\left(\lambda_{\mathrm{rf}}\right)=\frac{F_{0}}{1+{\left(\frac{\lambda_{\mathrm{rf}}-\lambda_{0}}{\Delta \lambda }\right)}^{2}}$$ (1)

Fitting parameters are given in Table 1.

**Table 1.** Lorentzian fitting parameters for DOPC efficiency as a function of spectral detuning.

	*F*_0_ (%)	λ_0_ (nm)	Δλ (nm)
SLM/WFS conjugated to the achromatic plane			
*L* = 250 μm	11.4	665	126
*L* = 500 μm	6.5	671	53.7
SLM/WFS conjugated to the guide star plane			
*L* = 250 μm	5.34	680	26.1
*L* = 500 μm	2.23	689	36.0

As expected, a larger spectral width is observed when conjugating the SLM/WFS to the achromatic plane for both samples, and the largest spectral width and peak energy are obtained for the thinnest sample. The maximum fraction of refocused energy is ≃12% for the *L* = 250-μm-thick sample and roughly half for *L* = 500 μm. Less expected is the fact that a larger focused energy is obtained at the achromatic plane than at the guide star plane, even at the guide star wavelength. We partially attribute the improvement of DOPC at the achromatic plane to the smaller density of optical vortices in this plane—roughly half the one in the guide star plane—potentially resulting in less phase reconstruction errors. Unexpectedly also, the peak of the focused energy distribution is slightly blue-shifted with respect to the guide star wavelength. This shift is in favor of one photon fluorescence excitation, but its explanation is not obvious (we ruled out a possible SLM calibration error), all the more because it differs between the cases where the SLM/WFS are conjugated to the achromatic plane (δλ ≃ 22 nm) or the guide star plane (δλ ≃ 5 nm). We experimentally checked that the DOPC spectral widths measured by detuning the playback laser line are consistent with both the speckle correlation width of the medium (fig. S11) and with the DOPC spectral widths measured by broadening the spectrum of the playback laser (fig. S13). Our results are thus relevant to nonlinear optical imaging modalities requiring multiline or broadband ultrashort laser beams.

### Required photon budget

**Fig. 4.**
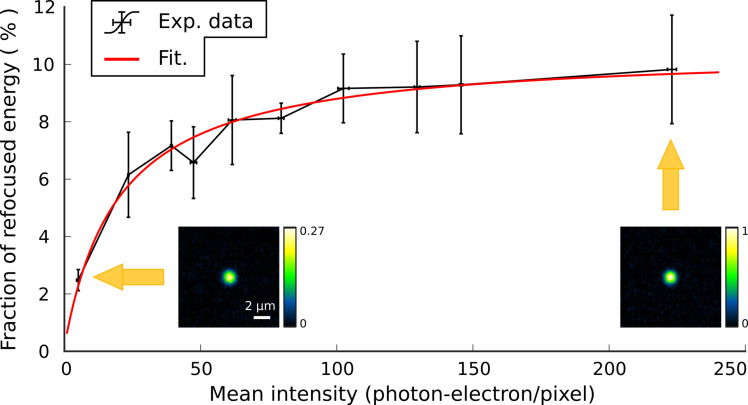
Efficiency of DOPC as a function of the guide star photon budget. The fraction of refocused energy is measured as a function of the average number of collected photon per camera pixels at the WFS and fitted. Here, the speckle patterns were generated using a 1° holographic diffuser located 3 mm away from the focused laser guide star at 635 nm. The SLM and WFS are conjugated to the virtual image of the guide star (like in Fig. 2). Vertical bars represent twice the experimental SDs (±σ) computed over five measurements.

The photon budget available from guide stars for performing DOPC is usually low either because of speed requirement or just because of the limited photon budget of the guide star itself. Furthermore, through a scattering medium, this energy is split among all outgoing spatial modes. Under these conditions, increasing the numerical aperture of the collecting objective lens (0.25 NA in our case) does not help since it does not increase the spatial-mode density of photon at the diffuser output, which solely matters. We thus investigated the photon budget required per spatial mode to perform WFS-based speckle phase conjugation. In Fig. 4, the fraction of focused energy *F* is plotted as a function of the mean speckle intensity at the WFS <*I*>, expressed in photoelectron per WFS pixel. The measurements are fitted with a theoretical model from (*59*): $F=F_{0}{\left(1+\frac{I_{0}}{<I>}\right)}^{-1}$ , with *I*_0_, the required average intensity to get half the optimal performances. Experimentally, we obtain that this model allows a good fitting of experimental data for a parameter *I*_0_ ≃ 20 photoelectrons per pixel. Considering that our DOPC system was designed to measure and control ≃3000 spatial modes with our 4-Mpx WFS camera, our measurements yield a required photon flux of ≃3 × 10^4^ photoelectons per spatial mode. We could also measure that the ratio between the playback speckle extent (without DOPC) and the playback focal spot size (with DOPC) is ≃62, giving a number of spatial modes for the diffuser equal to π/4 * 62^2^ = 3019, in excellent agreement with our optical design estimate.

### Single-shot DFPC from a fluorescent guide star

**Fig. 5.**
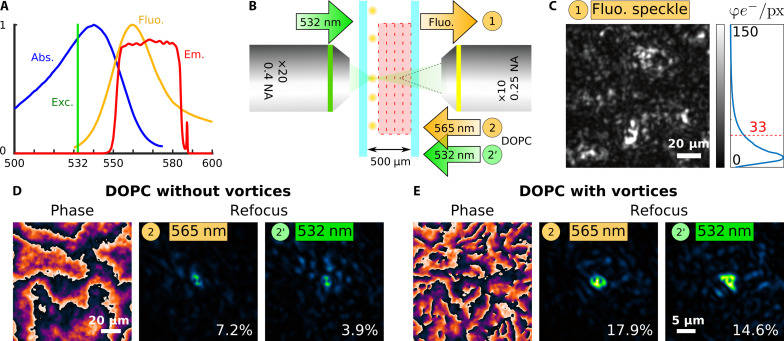
DFPC using 1-μm fluorescent beads as guide stars. The absorption (Abs.) and fluorescence emission (Fluo.) spectra of the beads are shown in (**A**) together with the excitation laser line at 532 nm (Exc.) and the band-pass emission (Em.) filter 565/30. Beads are hidden behind a thickness *L* = 500 μm of a paraffin samples (**B**). The SLM/WFS are conjugated to the achromatic plane of the scattering samples, and the measured fluorescence speckle is shown in (**C**) together with its intensity histogram exhibiting a mean fluorescence intensity of 33 photoelectrons per pixel. The playback laser beams is then sent through the sample after phase conjugation both at the average emission wavelength (λ = 565 nm) and at the excitation wavelength (λ = 532 nm) when considering the smooth phase contribution only (**D**) or the full reconstructed phase (**E**). The focii quality in (D) and (E) is degraded by the refractive index mismatch between the fluorescent microbead and the embedding PVA matrix.

Last, we demonstrate the ability of our system to perform single-shot DFPC using fluorescent micrometer-size spheres. Fluorescence excitation was achieved using a 532-nm laser line, and fluorescence was collected by stacking a pair of two emission filters to efficiently block the excitation light (Fig. 5A). The resulting filter bandwidth of 30 nm has been chosen so as to match as much as possible the spectral width of fluorescence, within the limit of the spectral correlation bandwidth of the scattering sample (Fig. 3B and fig. S11). The beads were spin-coated in a poly-vinyl alcohol (PVA) solution on a coverslip and then covered with four layers of parafilm (resulting in a 500-μm-thick scattering sample) and another coverslip on top (Fig. 5B). The excitation of the fluorescent beads was achieved by focusing the 532-nm laser from the rear of the sample with excitation intensity of <10 kW cm^−2^. Noteworthy, neither the power of our laser source (<5 mW) nor the single-photon fluorescence-absorption mechanism used allowed us to efficiently excite single isolated microbeads through the scattering medium. However, several solutions have been investigated to generate guide stars in-depth (*7*), especially using two-photon excitation (*6*, *11*). In fine, the total fluorescence photon budget at the WFS was 1.4 × 10^8^ photoelectrons, corresponding to a mean photoelectron budget per pixel equal to 33 (see Fig. 4). The SLM/WFS was conjugated to the achromatic plane of the scattering sample, and the fluorescence speckle signal was imaged and measured at the WFS (Fig. 5C). Last, the conjugate phase was addressed to the SLM without (Fig. 5D) and with (Fig. 5E) including optical vortices, and the playback laser beam was sequentially tuned to both the average fluorescence wavelength (565 nm) and the excitation wavelength (532 nm). In both cases, complete phase reconstruction outperforms the regular AO scheme only compensating for ballistic light, further demonstrating the ability to robustly mistune the playback laser wavelength to take into account the fluorophores Stokes’ shift at the expense of a slight loss in refocused energy (14.6 versus 17.9%). When conjugating the SLM/WFS to the guide star plane, no focus could be obtained. Noteworthy, the focus quality obtained with fluorescent microbeads (Fig. 5) is noticeably degraded as compared to all other focii we could obtain in all other conditions, including for spectrally broad or spectrally shifted readout lasers (see fig. S14). The focii degradation observed in Fig. 5 (D and E) is due to the presence of the fluorescent microbead on the beam path because its refractive index is not matched with the one of the PVA embedding matrix (*17*). Moreover, it was not possible to shift the playback focus away from the microbead without inducing important focus-energy degradation because the isoplanatic patch of our sample is typically comparable to bead size itself. To characterize the DFPC performances, we thus measured the fraction of playback light energy in the main focus as described in fig. S15. Performances are quantified in Table 2 for three samples thicknesses.

**Table 2.** DFPC average efficiency (± experimental SDs) for a playback laser wavelength λ_rf_ at the average emission wavelength (λ_rf_ = 565 ± 2.5 nm) and at the excitation wavelength (λ_rf_ = 532 nm) from fluorescent light emitted by microbeads (λ_rf_ = 565 ± 15 nm) as a function of paraffin thickness.

	*L* = 250 μm	*L* = 375 μm	*L* = 500 μm
**DFPC at the average fluorescence wavelength (λ_rf_ = 565 nm)**			
With vortices	11.3 % (± 5.8)	9.3 % (± 4.1)	7.1 % (± 6.1)
Without vortices	6.8 % (± 2.3)	3.9 % (± 0.9)	3.7 % (± 2.1)
**DFPC at the excitation wavelength (λ_rf_ = 532 nm**)			
With vortices	8.7 % (± 3.3)	9.5 % (± 4.7)	6.5 % (± 4.8)
Without vortices	4.7 % (± 1.7)	2.8 % (± 1.5)	2.3 % (± 1.2)

## DISCUSSION

In this work, we have demonstrated the ability to perform single-shot DOPC from incoherent guide stars hidden behind forward scattering samples. This progress has been made possible thanks to two conceptual improvements: the ability to rebuild complex speckle patterns with a high-resolution WFS on the one hand and the large spectral bandwidth revealed by scattering media with large anisotropy factors on the other hand. Our results not only underline the importance of rebuilding optical vortices that have typically been ignored by Shack-Hartmann WFS users but they also prove the key role of associated spiral phase structures for DOPC performances. We also demonstrate the ability to use fluorescent microbeads as guide stars, which are associated with three issues: the weak fluorescence photon budget challenging the maximum number of modes one may rebuild, the ≃30-nm spectral bandwidth of the fluorescence signal, and the Stokes’ shift between the fluorescence and the excitation spectra. The spectral width of DOPC efficiency through 500-μm-thick paraffin samples is characterized and qualitatively matches the spectral correlation width of the medium. The spectral width of the scattering medium is driven by the SD of optical path length trajectories and imposes an upper limit for the spectral width of the emission filter. Beyond this limit, the speckle cannot be considered as achromatic, and only an averaged wavefront can be defined, wherein optical vortices are scrambled, reducing the focusing ability of the AO system. In this regard, we also demonstrate the importance of conjugating the WFS and the SLM with the achromatic plane of the scattering slab by demonstrating the degraded performances when conjugation is achieved with the guide star plane. In the end, the emission filter width is a compromise between the contrast of the fluorescence speckle at the WFS and the photon budget. We estimated that our solution requires an average photon budget per spatial mode of the scattering medium of the order of 3 × 10^4^.

In AO systems, the SLM/WFS are often placed in the Fourier plane. Our system did not allow us to easily switch from the sample plane to the Fourier plane, and a fair comparison might deserve further experimental characterization. As an argument in favor of our configuration, in the Fourier plane, the beam scattered by a forward scattering medium experiences a transverse homothetic dilation for a wavelength shift, so that the maximum number of measurable/controllable modes is limited by ${\left(\frac{\lambda }{\delta \lambda }\right)}^{2}$ . In contrast, the achromatic plane is the origin of the longitudinal axial dilation responsible for the transverse dilation at infinity (*49*, *51*). No such transverse dilation occurs, and the speckle correlation is solely limited by the intrinsic spectral correlation width of the scattering medium. Consequently, for broadband light manipulation, the number of modes we can manipulate is not limited in the achromatic plane, in contrast to the Fourier plane where fluorescence speckles are blurred. This Fourier-conjugated configuration was in particular chosen by the authors of (*23*), where a 10-nm filter was used to increase the coherence of the fluorescence signal and improve the number of measurable modes. Further quantitative comparison with the technique (*27*) would be deserved, especially regarding the DOPC performances as a function of the nature of phase distortions (including low-order aberrations or not) as well as the photon budget per mode.

Noteworthy, provided that our technique enables efficient multiple-scattering compensation through samples, in particular those leaving no ballistic light, the guide star size should not exceed the autocorrelation width of the original speckle pattern. Besides the technical challenge to generate isolated guide stars, especially in densely labeled samples, the isoplanatic patch size associated with the obtained readout focus appears as critically small for imaging applications. This intrinsic constraint imposed by such samples makes our achievement more relevant to applications such as in-depth photostimulation, thermal phototherapy, in-depth caged drug photo delivery, light-induced guided neuronal growth, nonimaging cell activity monitoring, or microendoscopy through fiber bundles. Brain cells monitoring through the skull also appears as especially interesting thanks to the distance separating the scattering medium (the skull) from the object of interest (the brain), yielding an isoplanatic patch size of ≃15 μm (*14*, *63*).

Besides opening a solution for performing DOPC, this work motivates further fundamental investigations about forward scattering media. First, we could not attribute the spectral blue-shift observed in Fig. 3 to a miscalibration of our DOPC system. The difference in the amount of blue-shift between the two sample thicknesses (*L* = 250 μm and *L* = 500 μm) and between the two imaging conditions (conjugation with the guide star plane or the achromatic plane) suggests a physical reason for blue-shifted light to yield larger DOPC efficiency than at the guide star wavelength. Blue-shifted light may benefit more from phase conjugation because of the multiple-scattering process scales as λ^2^ in forward scattering media (*49*) [in contrast to the λ^4^ scaling in the Rayleigh scattering case (*4*)]. Second, by contributing to bridging the gap between AO and wavefront shaping, this work about broadband forward scattering media interrogates about the relevance and the possibility to define a theoretical discrimination criterion between optical aberrations and light scattering. The distortion matrix concept has supported this distinction (*53*). Usage has typically used the term aberrations for low-order wavefront distortions only although high-order smooth distortions exhibit no fundamental difference. However, the presence of optical vortices in the wavefront suggests making out from wording aberrations since optical vortices necessarily originate from at least three interfering beams (*64*) and thus suppose several optical trajectories.

## MATERIALS AND METHODS

### Sample preparation

The 720-μm acute slice of spinal cord of a 6-day mouse was fixed in paraformaldeyde and mounted in fluoromount between two coverslips separated by an 800 spacer. Nail varnish was used to minimize evaporation. The sample was left vertically in place for 24 hours for gravity drifting to stop.

Parafilm samples were prepared by stacking two, three, or four layers of parafilms between two coverslips and gently pressed to minimize air spacing and thus surface scattering at interfaces. The thicknesses of the stacks were measured thanks to a calibrated translation stage by observing the back reflexion of the guide star on the internal coverslip surfaces right aside from the sample. To ensure isotropic scattering, the parafilm layers were oriented at 90° for the two-layer samples and at 0°, 45°, 90°, and −45° for the four-layer samples.

The bead samples were prepared by stacking parafilm diffusers onto a spin-coated layer of beads embeded in PVA. The layer was prepared by spin coating at 10,000 rpm, a 10^−4^ weight solution of 1-μm fluorescent microbeads (Fluospheres F8820, Thermo Fisher Scientific) in a 2% weight solution of PVA. For the two-parafilm-layer sample, two #1 coverslips were used to add a ≃500 μm spacing between the layer of beads and the parafilm.

### Optical setups

A detailed description is provided in the Supplementary Materials. Briefly, two optical systems were used, a first one for comparing speckle field reconstruction by wavefront sensing and digital holography (DH) (see fig. S1) and a second one to perfom DOPC through scattering samples using a WFS (fig. S5). To obtain Fig. 2, the first objective lens (Obj. 1) was removed to illuminate the sample with a collimated laser beam. In the DOPC experiments, the SLM and WFS were conjugated to the so-called achromatic plane, located in a virtual plane lying at a depth equal to two-thirds of the sample thickness (or equivalently, the fluorescent guide star depth). The location of the output plane of the scattering sample was first identified thanks to white light illumination, and the objective was then moved forward of the right amount toward the scattering sample.

In the DOPC experiment, the guide star was generated by using a focused laser beam originating from fiber-coupled diode laser (532 and 635 nm) or a supercontinuum laser (ElectroVis-470, LEUKOS, France) combined to a computer-tunable filter box (Bebop, LEUKOS, France). The laser was focused into the sample with a ×20, 0.4 NA objective lens, and the scattered light was collected using a ×10, 0.25 NA objective lens.

In the fluorescent guide star experiment in Fig. 5, the microbeads were excited from the rear of the scattering medium using a focused 532-nm laser. The excitation intensity was <10 kW cm^−2^, and the exposure time at the WFS was 1 s.

The WFS design is similar to the one described in (*32*). It is made of a two-dimensional 20-μm-step checkerboard 0 − π-phase grating conjugated to a CMOS camera (BSI Prime Express, Photometrics) thanks to a relay telescope. An additional −75-mm diverging lens was fixed at the C-mount thread of the camera to minimize field curvature introduced by the relay telescope. The final global magnification of the imaging relay system was ≃3.

In the first optical system comparing DH to wavefront sensing (fig. S1), a 635-nm diode laser was spatially filtered by a monomode fiber and collimated to illuminate a 1° holographic diffuser (Edmund Optics). This diffuser was tuned out-of-focus the WFS, and an iris was placed in an intermediate Fourier plane to tune the speckle grain size. For DH experiments, the grating of the WFS was removed, and a reference arm generated from a beam splitter was placed before the scattering sample. The reference beam and the “signal” speckle beam were combined together with an angle larger than the opening angle of the speckle, thanks to a pellicle beam splitter located in the Fourier plane of the relay telescope of the WFS.

Last, a polarizer was added in the optical path of the WFS because scattering media, even forward scattering, slightly depolarize light beams (*65*). In our case, we measured that, for a thickness *L* = 500 μm of paraffin, the polarization rate was 0.83. This slight depolarization thus accounts for ≃17% of degradation in DOPC performances since our SLM can only modulate a single polarization component.

## Supplementary Materials

### This PDF file includes:

Supplementary Text Figs. S1 to S15 References
