# Supplementary material for: Single-shot digital optical fluorescence phase conjugation through forward multiple-scattering samples
Source: Sci Adv. 2024 Jan 19;10(3):eadi1120. doi: 10.1126/sciadv.adi1120 (PMC10798569; doi:10.1126/sciadv.adi1120)
Supplement: Supplementary file 1 — Supplementary Text Figs. S1 to S15 References [file sciadv.adi1120_sm.pdf]

Science Advances
AAAS

Supplementary Materials for

Single-shot digital optical fluorescence phase conjugation through forward multiple-scattering samples

Tengfei Wu *et al.*

Corresponding author: Marc Guillon, marc.guillon@u-paris.fr

Sci. Adv. **10**, eadi1120 (2024)
DOI: 10.1126/sciadv.adi1120

This PDF file includes:

Supplementary Text
Figs. S1 to S15
References

S1 Quantitative measurement of complex field with a wavefront sensor

S1.1 Experimental setup

In this section, we experimentally demonstrate that our wavefront sensor (WFS) can quantitatively measure a scattered speckle field from a single-shot acquisition by comparing the retreived phase and amplitudes to the one obtained by off-axis digital holography (DH), which we consider as a ground truth. Fig. S1 shows the full experimental setup, in which the collimated beam with a wavelength of $635\ \mathrm{nm}$ passes through a polarized beam splitter, separated into the reference beam path and the signal beam path. In the signal beam path, the beam interacts with a 5° holographic diffuser (Edmund Optic), and the complex field is imaged on the WFS by a telescope with a lens set of f=$100\ \mathrm{mm}$ and f=$200\ \mathrm{mm}$, respectively. In order to tune the speckle density, an adjustable diaphragm is placed in the intermediate Fourier plane of the telescope. The WFS is composed by a two-dimensional $20\ \mu\mathrm{m}$-step checkerboard phase grating, placed $3\ \mathrm{mm}$ away from the camera sensor, a telescope with a lens set of f=$100\ \mathrm{mm}$ and f=$200\ \mathrm{mm}$, and a divergence lens with a focal length f=$-50\ \mathrm{mm}$, which is used to correct the field curvature. A beam block (ZBB) is placed closed to the intermediate Fourier plane of the telescope in the WFS, to only select the first diffracted order of the grating pattern (blocking the zero order and higher orders). In the reference beam path, a set of mirrors are used to adjust the length of the optical path and the tilt angle between two beams to optimize the contrast of interference fringes in DH measurement. A lens with a focal length f=$50\ \mathrm{mm}$ and a pellicle beam splitter is used to image the reference beam on the camera. Two half waveplates are placed respectively in the two paths to adjust the relative beam intensity. A beam blocker BB is used to collect the useless beam in the experiments. In the data acquisition step, for both methods we take two measurements with and without scattering sample and compare the relative complex field between the two measurements for each method. In order to be sure that the two methods measure the same scattering signal, we first measure the field without scattering sample in DH (i.e. open both the two paths and remove the grating G and beam blocker ZBB). Then we insert the scattering sample and measure the scattering field in DH. After finishing the measurement in DH, the reference beam path is blocked and we place the grating G and beam blocker ZBB to do the measurement with scattering sample in WFS. Finally, we remove the scattering sample to do another measurement in WFS. 50 frames are recorded and averaged for each measurement for better signal to noise ratio.

S1.2 Phase gradient measurement from a lateral shearing interferometer WFS

Off-axis DH can directly measure a complex field (phase and amplitude) by extracting the first order of the fringe pattern in the Fourier space. In contrast, the WFS does not measure directly the phase but the phase gradient. Here we describe in detail the procedures to extract the phase

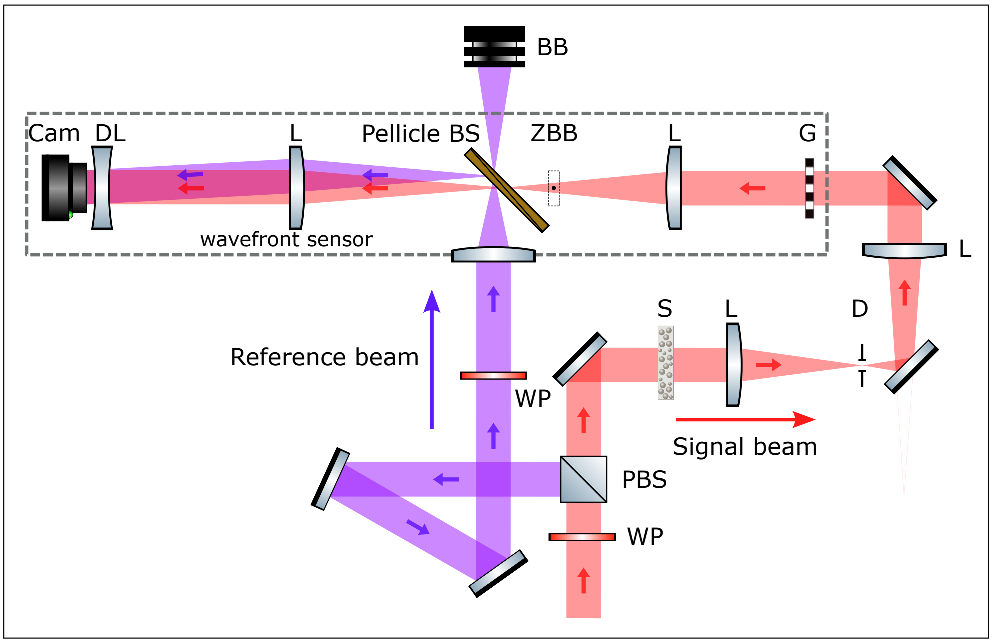

Figure S1: **Full experimental setup of the demonstration of the quantitative measurement of complex field by a lateral shearing interferometer WFS.** WP: Half wave plater; PBS: Polarized beam splitter; S: Scattering sample; L: Lens; D: Diaphragm; G: Grating; ZBB: zero-order beam blocker; BB: Beam blocker; Pellicle BS: Pellicle beam splitter; DL: Divergence lens; Cam: Camera

gradient information from the raw camera image of a quadriwave lateral shearing interferometer (QWLSI) (*33, 34*). A detailed description of the full gradient recovery process from QWLSI measurements is given in Ref. (*66*). Fig. S2 briefly summarizes and illustrates the main steps.

S1.3 Measurement of complex scattering field WFS vs DH

In Fig. S3, we compare the complex field reconstruction obtained from both the WFS and off-axis DH, thanks to the optical system shown in Fig. S1. In these images, the number of vortices in the beam at the WFS (*i.e.* the number of spatial modes) was 1581. The intensity image in the DH part is obtained by blocking the reference beam for direct intensity recording. Both the phase difference and intensity difference are shown, demonstrating the good agreement between WFS and DH measurements. A remaining low-spatial frequency content appears in the phase difference pattern, probably due to cumulative integration errors of phase gradients estimates over the large number of modes.

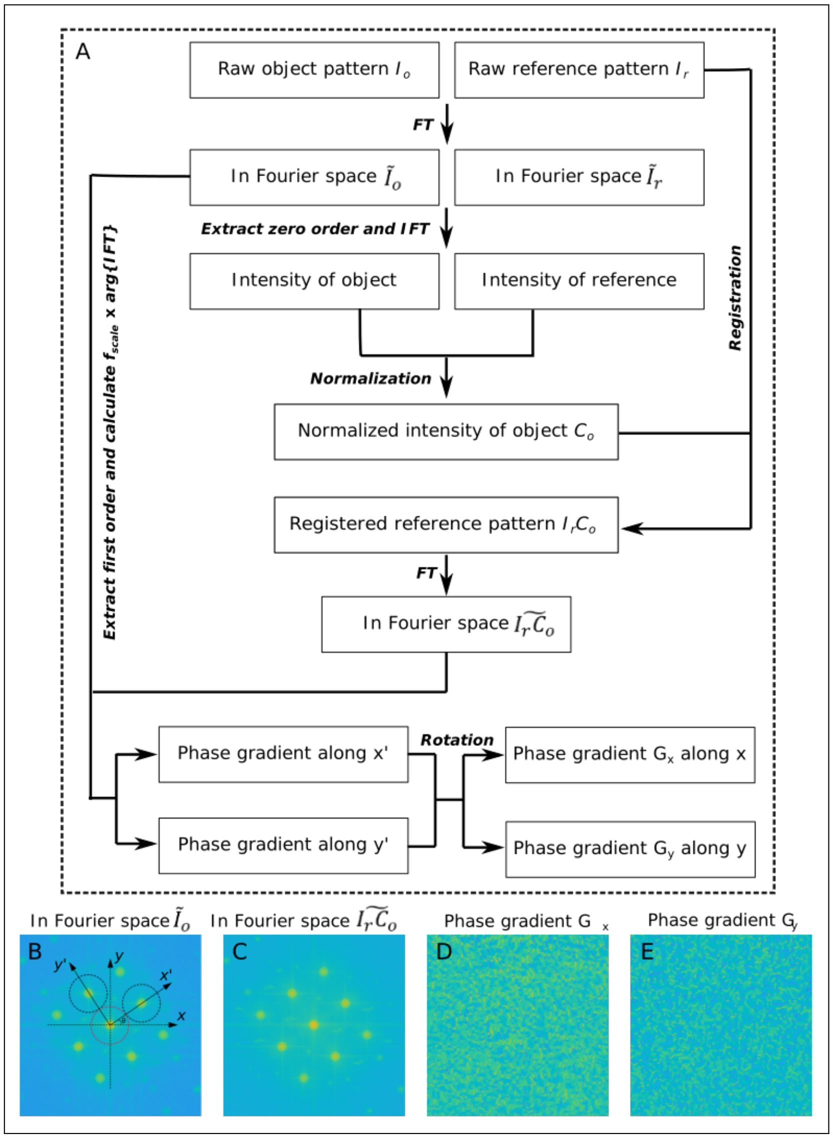

Figure S2: **Phase gradient reconstruction procedure from a quadri-wave lateral shearing interferometer WFS.**

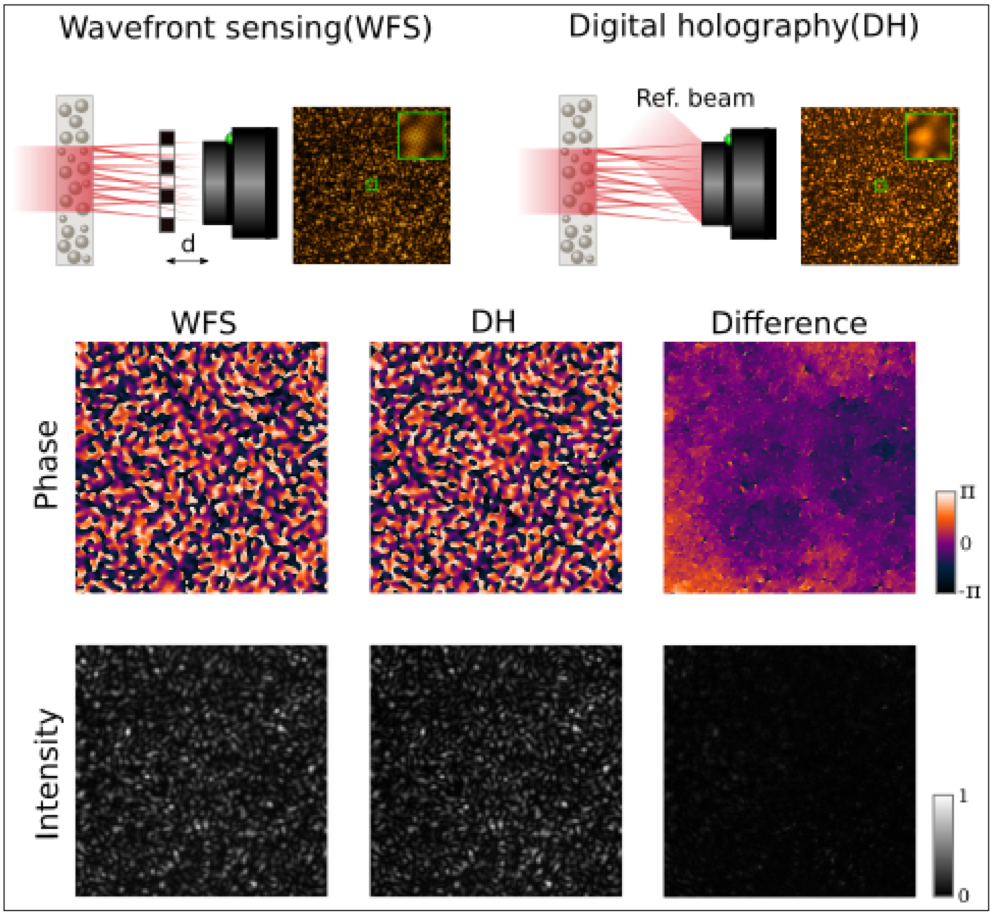

Figure S3: **Comparison of speckle field reconstruction using a lateral shearing interferometer WFS and off-axis digital holography (DH).**

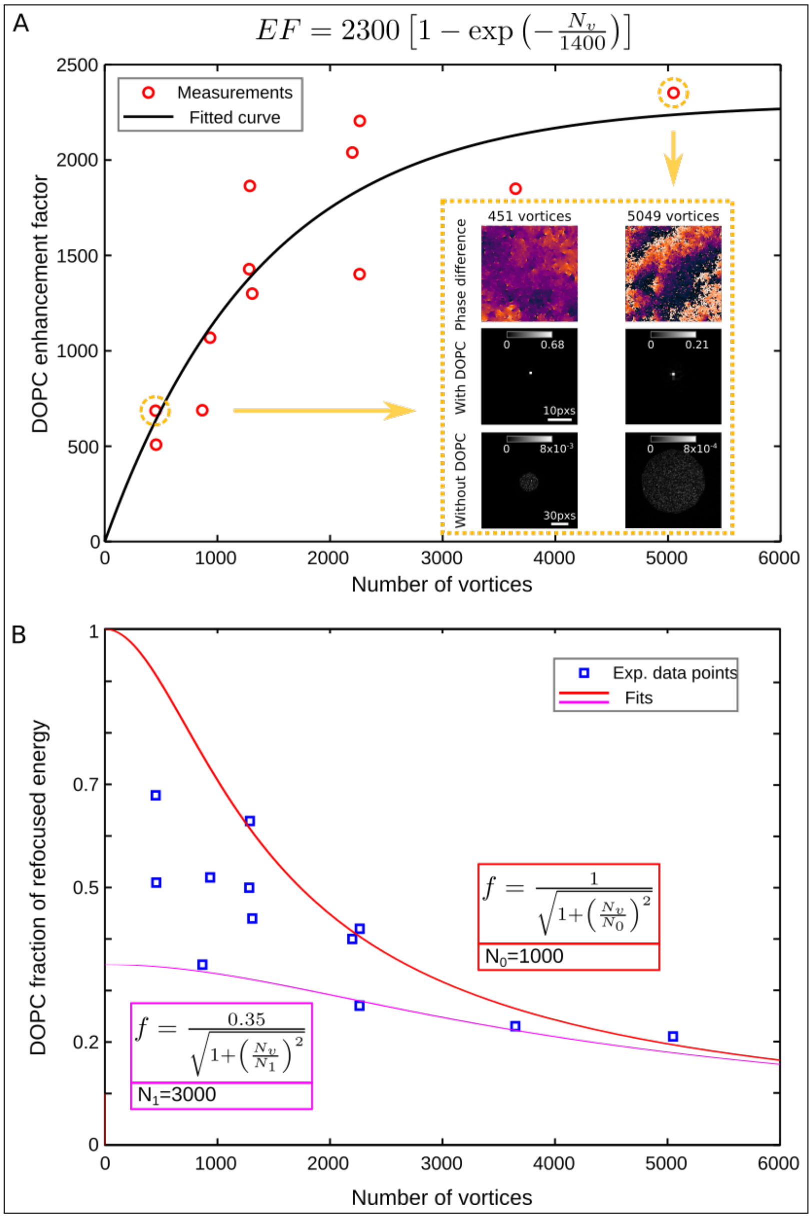

Figure S4: **Experimental estimation of the maximum number of modes that our WFS can rebuild to achieve DOPC.** The number of vortices (*i.e.* the number of modes) is varied by tuning an iris diaphragm in a Fourier plane (D in Fig. S1). The phase is measured with the WFS and compared to the phase measured by DH, the latter phase pattern being considered as the ground truth. Then, digital optical phase conjugation (DOPC) is numerically simulated based on the phase difference, both to compute the enhancement factor (A) and the fraction of refocused energy (B). Experimental data points are fitted using empirical curves to underline and quantify the global trend. As expected, the slope of the DOPC for small number of modes is close to one (*10, 59*).

S2 Experimental setup and calibration for digital optical phase conjugation with a wavefront sensor

S2.1 Experimental setup

In this section, we mainly show the detailed experimental setup for performing the digital optical phase conjugation (DOPC) with a WFS. A collimated guide-star (GS) laser beam passes through a pellicle beam splitter (PBS) and an objective Obj.1 (x20, 0.4NA) focuses the beam that generates a guide-star (or excites a fluorescent micro-bead guide-star) at the rear of the scattering sample S. The scattered field is imaged onto the SLM by an afocal telescope, which is composed by an objective Obj.2 (x10 0.25NA), a diverging lens DL with a focal length f=-50 mm and a wavefront correcting lens with a focal length f=1000 mm placed in front of the SLM. As compared to a regular Galileo telescope made of two converging lenses in a 4-f configuration, our system has the advantage to allow large magnification ($\times 110$) between the sample plane and the SLM plane over a shortened physical distance. Then, a telescope with a lens set of f=300 mm and f=150 mm is used to conjugate the SLM plane to the WFS, which has the same components as the previous experimental setup described in Sec.S1.1. A removable mirror (not shown) can be placed between the pellicle beam splitter and the WFS grating G to enable the recording of the reference pattern. After measuring the scattered field, the conjugated phase is loaded on the SLM. A collimated ”playback” laser beam is reflected by a polarized beam splitter and propagates back to the SLM. The linear polarizer P, used to polarize the beam originating from the scattering sample is also used to clean the polarization of the ”playback” laser beam and match the modulating polarization axis of the liquid-crystal SLM. The wavefront modulated by the SLM then passes through the scattering sample. The focus of the ”playback” laser beam at the guide-star position is monitored on a camera through Obj.1 thanks to a tube lens TL (f=150 mm).

S2.2 Optimization of the distance between the grating and the camera

For a lateral shearing interferometer WFS, the grating-camera distance *d* drives a trade-off between the phase sensitivity, phase gradient dynamic and spatial resolution. In our case, spatial resolution is of critical importance to measure complex wavefields containing as many spatial modes as possible. The number of modes roughly equals the number of optical vortices of a fully developed speckle pattern. Fig. S6A shows the optical vortex distribution in a measured complex scattering field. The yellow and blue peaks denote the random locations of vortices of charges $+1$ and -1, respectively. ΔA stands for the Laplacian of the vector potential A, which account for the solenoidal contribution in the Helmholtz decomposition of the phase gradient vector field measured at the WFS, as detailed in Ref. (*46*). ΔA yields peaks (in theory Dirac functions) at vortices locations. Segmentation splits the ΔA image into multiple small regions according to the locations of the peaks. A following integration within each small region gives the specific vortex charge in each location, according to Stokes' theorem (*46*). The histogram

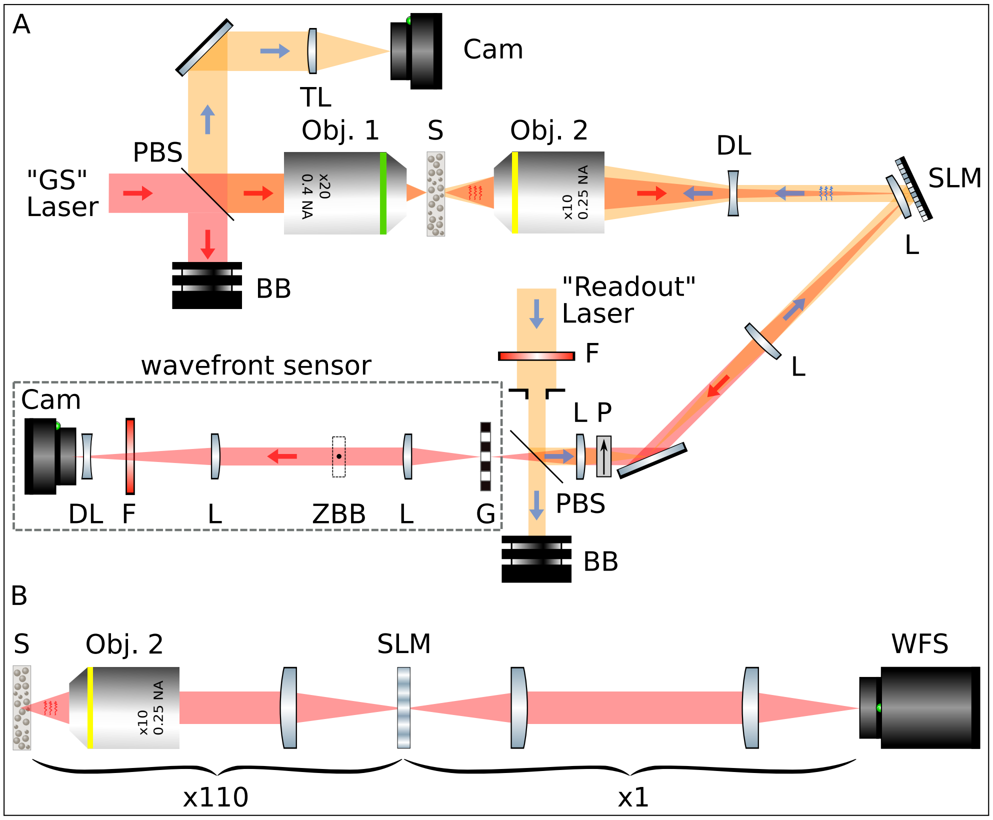

Figure S5: **Full and equivalent experimental scheme of the optical phase conjugating microscope.** A) Full experimental system. PBS: Pellicle beam splitter; BB: Beam block; S: Scattering sample; DL: Diverging lens; L: converging lens; SLM: Spatial light modulator; P: Polarizer; G: grating; ZBB: Zero-order beam block; F: spectral filter used for fluorescence experiment but removed otherwise; Cam: Camera; TL: Tube lens. B) Equivalent representation of the speckle beam control arm. The configuration in A) allows having a large magnification imaging system with reduced size on the optical table.

of the distribution of the estimated charges of the optical vortices in the complex scattering field is shown in Fig. S6C, in which three peaks occurs at charge +1/-1 and 0. Here we use the histogram contrast, which is defined by the ratio between the width of the central peak w and the distance L between the central peak and the side peak, to evaluate the vortex-discrimination ability of the WFS. The ratio $\frac{w}{L}$ is then plotted as a function of the distance d. The optimal distance d is finally obtained by computing the minimum coordinate of a parabolic fitting curve.

S2.3 Alignement and calibation of SLM and WFS

In order to compensate the scattering field in the DOPC experiments, a proper alignment and calibration of SLM and WFS is highly required. Optical elements conjugating the SLM to the WFS may induce some geometric transformation such as translation, rotation, and dilation between the SLM and WFS plane. In our experiments, we calibrate alignment issues thanks to a custom-designed vortex array. Specifically, we design a 20x20 vortex array with alternating $+1$ and -1 charges. Four additional symmetry-breaking vortices were added to the array to eliminate symmetry ambiguity problems. The grid thus contain 404 optical vortices in total. A phase map corresponding to this optical vortices array is shown in Fig. S7A. This pattern is loaded on the sub-region of the SLM lying in the field of view of the WFS. A collimated beam then illuminates the SLM and the reflected beam is then measured by the WFS. The ΔA map is then computed and compared to the generated vortex pattern. The histogram of the measured optical vortices is shown as an inset in Fig. S7A. The calibration of the SLM is achieved by centering the peaks of the vortex charge histogram on ± 1 values. A typical comparison between a still non-aligned measured vortex location and the loaded true vortices array is shown in Fig. S7B. To correct this mismatch, we evaluate the geometric transformation of the two vortices arrays by using the built-in function in Matlab ("imregtform", Matlab Image Processing Toolbox). With the evaluated transformation coefficients, we introduce a numerical correcting transform to the pattern measured by the WFS to align the two patterns as shown in Fig. S7C. We then compute the phase difference between the loaded phase map (in Fig. S7A) and the phase map measured by the WFS in Fig. S7D, where all phase vortices perfectly cancel out. This flat phase pattern ensures the possibility to achieve phase conjugation.

S3 Combination of DOPC and computer generated holography

In this section, we demonstrate two more DOPC related experiments with the measurement of the complex scattering field by WFS. We show that as long as the scattering is properly compensated, one can deliver a specifically designed hologram through the scattering sample. The experimental setup presented in Sec.S2.1 is used. The scattering sample here is a 1° holographic diffuser (Edmund Optics), ensuring a sufficient memory effect for the hologram delivery. The collimated beam with a wavelength of 635 nm passes through the object Obj. 1 and forms a

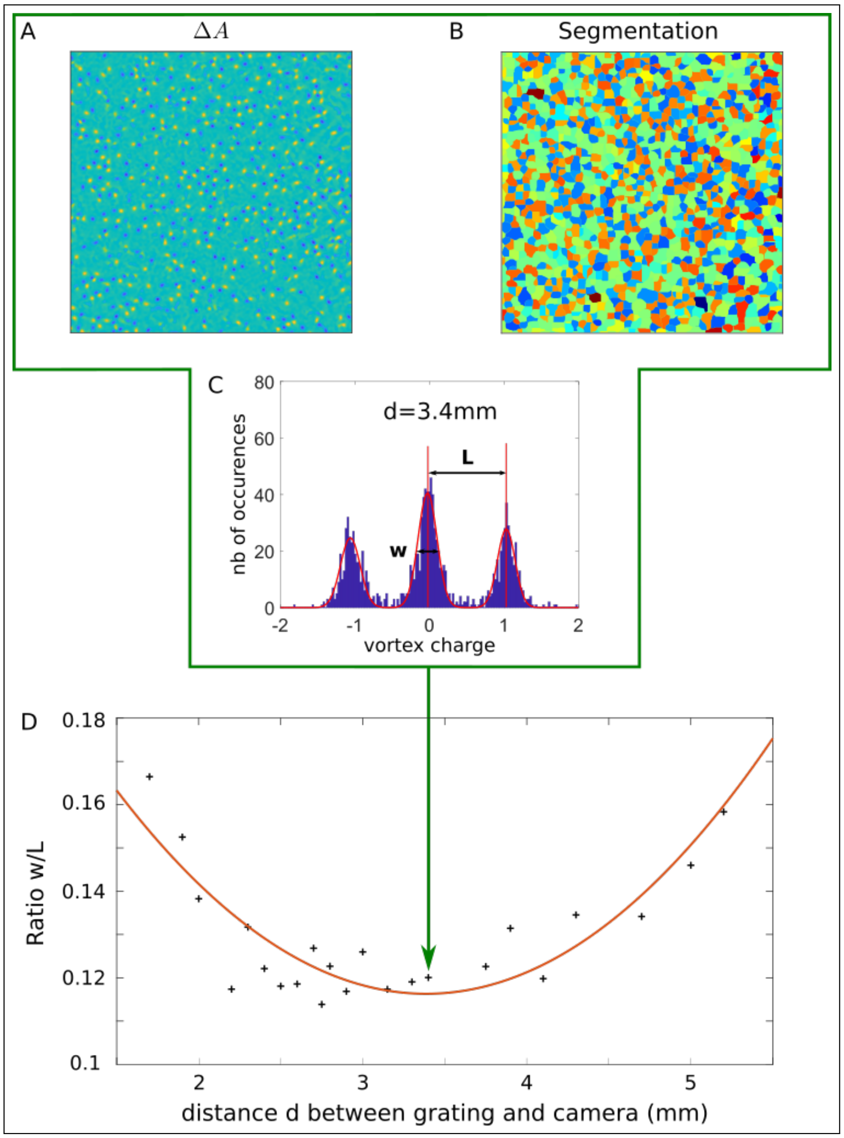

Figure S6: **Optimization of the distance grating-camera in the WFS for speckle patterns measurement.** (A) Laplacian of the vector potential shows the location of the optical vortices in a speckle field. (B) Image segmentation identifies the optical vortices map for topological charge measurement. (C) Histogram of the measured charges of optical vortices. The optimized grating-camera distance can be determined by the contrast of the histogram, defined as the ratio w/L where w is the peak width and L the distance between peaks. (D) Plot of the measured contrast as a function of grating-camera distance.

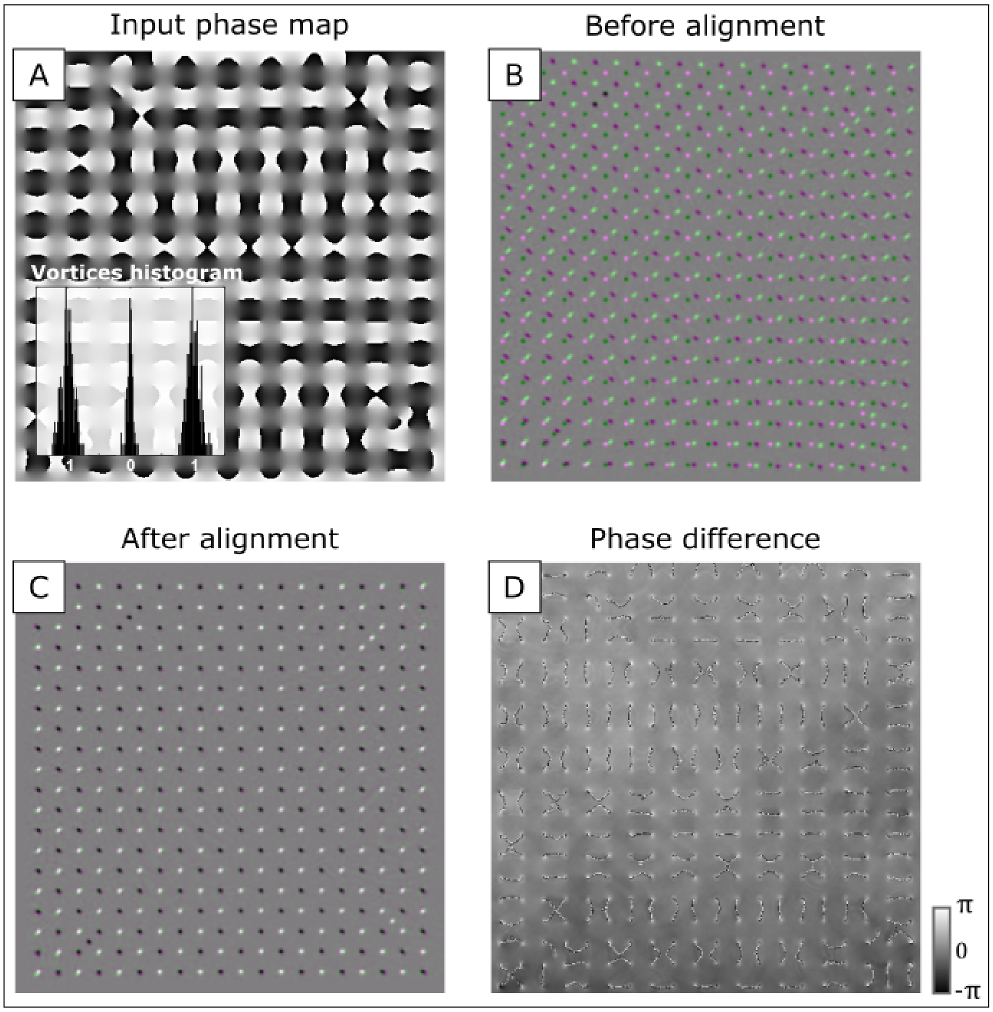

Figure S7: **Alignement and calibation of the SLM and WFS.** (A) Input phase map generated by a specific vortices array, used to align the mismatch between the SLM and the WFS plane. Inset is the histogram of the measured optical vortices. (B) Merged vortices maps of the input and the direct measurement from the WFS before alignment. (C) Merged vortices maps of the input and the measurement after alignment. (D) Phase difference between the input phase map and the measured phase map with alignment.

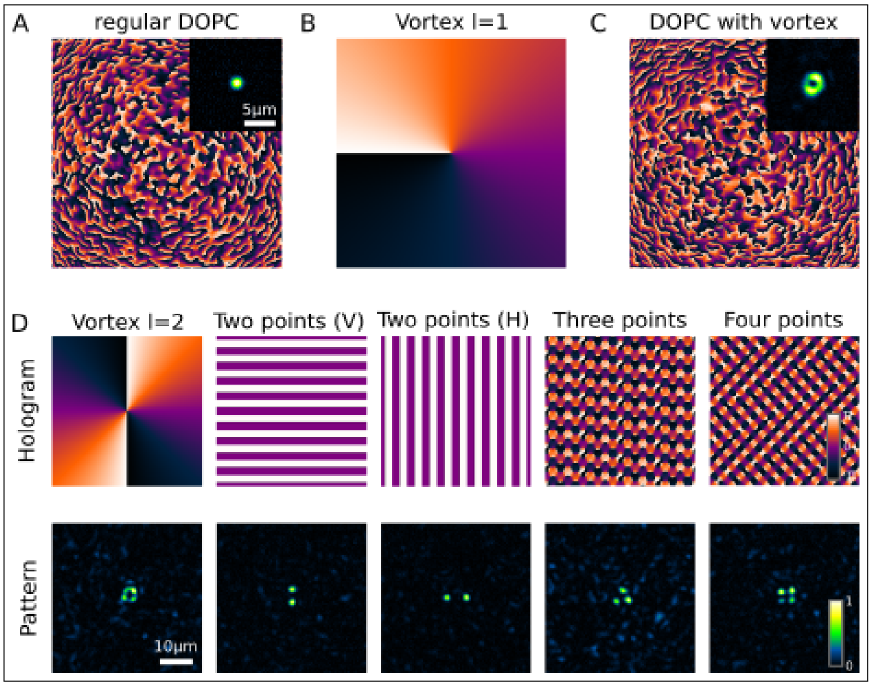

Figure S8: **Computer generated holography combined with DOPC for patterned excitation.** (A) The phase map used to perform DOPC through a 1° holographic diffuser. Inset is the focus after DOPC. (B) Hologram pattern for generating +1 charge optical vortex. (C) The resulted phase map for delivering a donut (+1 charge information) through the scattering sample. Inset is the result with DOPC. (D) Hologram patterns and corresponding focused patterns through the diffuser.

virtual guide-star, locating 3mm away from the diffuser surface. The SLM and WFS are conjugated to the guide-star plane. After measuring the complex scattering field, DOPC is performed through the scattering sample, as is shown in Fig. S8A the phase map and the refocus. In the next step, by adding another -1-charged optical vortex phase map (Fig. S8B) into the measured DOPC phase pattern, instead of only having a focus, we obtain a donut-shaped optical vortex beam on the camera, as is shown in (Fig. S8C). Similarly, other hologram patterns can also be delivered through the scattering sample with the measured complex scattering field. Several computer generated holograms could then be generated using a Gerchberg–Saxton algorithm (*67*). The delivery results are respectively shown in Fig. S8D. Such hologram pattern delivery can be interesting for e.g. the application of photostimulation.

S4 DOPC through a multimode fiber

This section aims at demonstrating the potential of our DOPC approach to refocus a laser beam through a multimode fiber. Specifically, we use a $1\ \mathrm{cm}$ long multimode fiber (FG050UGA,

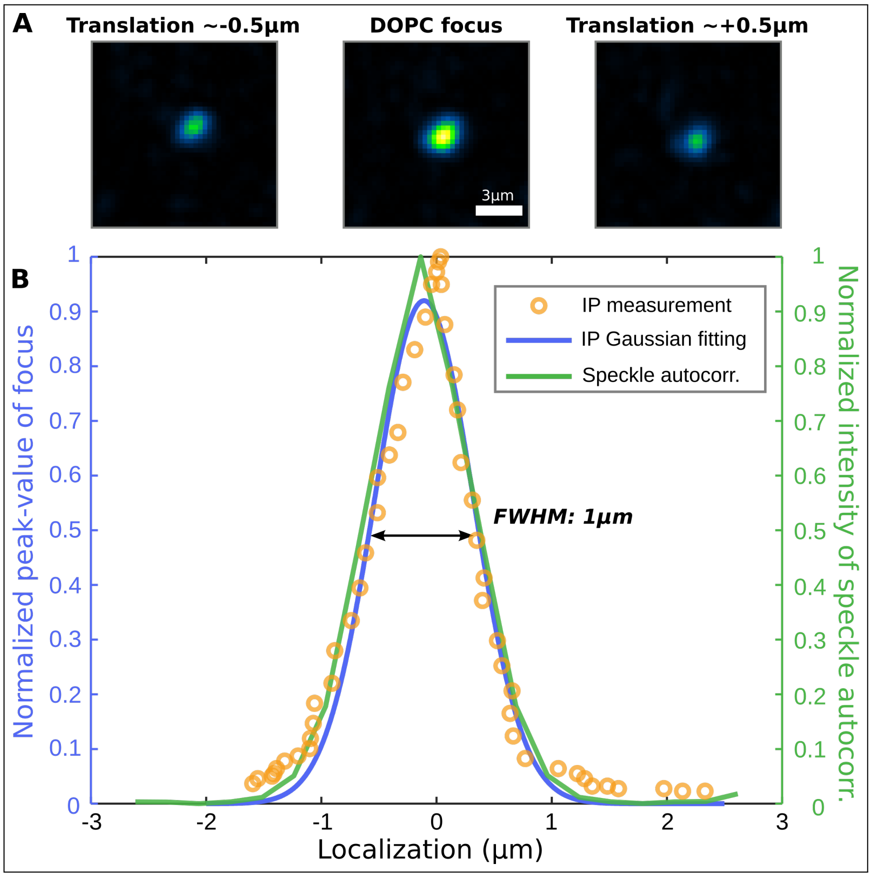

Figure S9: **Size of the isoplanatic patch (IP) through the $720\ \mu\mathrm{m}$-thick spinal cord slice in Fig. 1.** Phase conjugation yields a sharp focus that can be translated by shifting the pattern aside at the SLM (A) and its intensity decreases away from the guide-star location (A). The evolution of the peak intensity of the focus with the amount of translation is plotted in (B) (hollow orange circles) and compared to the autocorrelation plot profile of the speckle pattern measured in the absence of phase conjugation (green curve). The ability to translate the focused spot defines the dimensions of the isoplanatic patch (IP). The matching between the two curves demonstrates that phase conjugation effectively compensates multiple scattering events through the slice.

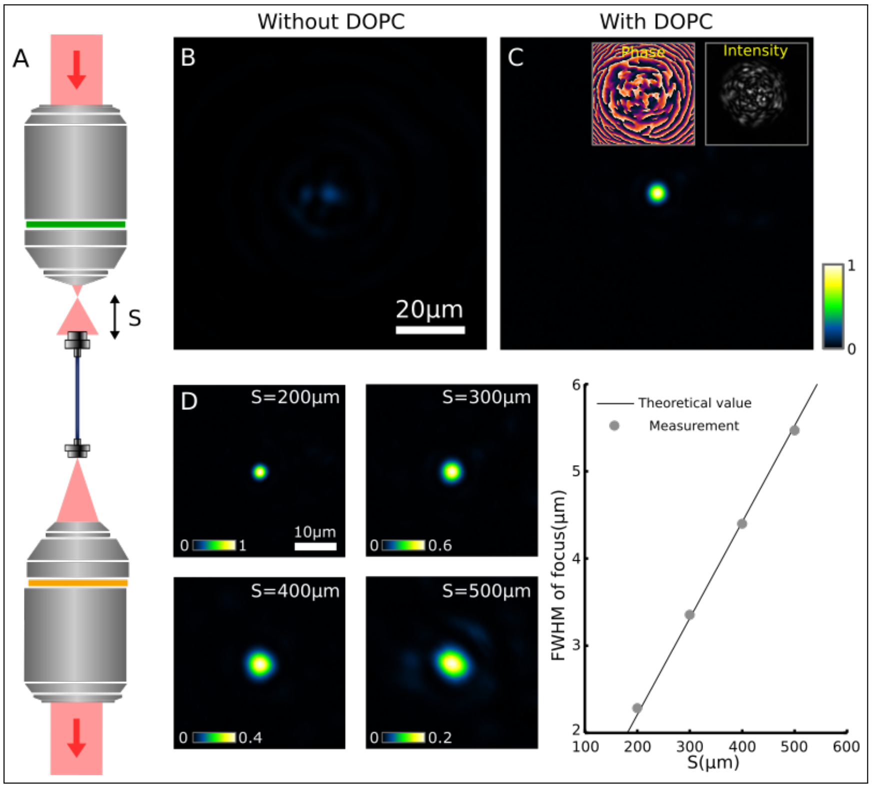

Figure S10: **DOPC through a multimode fiber.** (A) Schematic of the experiments. (B) The recorded image on the monitoring camera without DOPC. (C) DOPC results at S = 300 μm with the complex field measured by WFS. Insets are respectively the phase and intensity. (D) DOPC results for various distances of S and the plot of the size of the focus as a function of S.

thorlabs), and the virtual guide-star is generated at S = 300 μm away from the input fiber facet, as is shown in Fig. S10A. The output facet is conjugated to the SLM and WFS and the complex field is measured by WFS. We show in Fig. S10B the playback camera image with flat phase displayed at the SLM, showing a speckle intensity. The image after DOPC is compared in Fig. S10C, which gives a more intense focus on the camera (same colorbar). The complex field measured by the WFS is shown in Fig. S10C as insets. By translating the guide-star to different axial positions from S = 200 μm to S = 500 μm, we implement DOPC on all the cases, as we show in Fig. S10D. We can notice that the peak intensity of the focus decreases with increase of S, while the size of the focus (defined as the full-width- half-maximum) gets larger, and it follows the relation of $\sim \frac{\lambda S}{D}$, which is shown as a fitting plot in Fig. S10D.

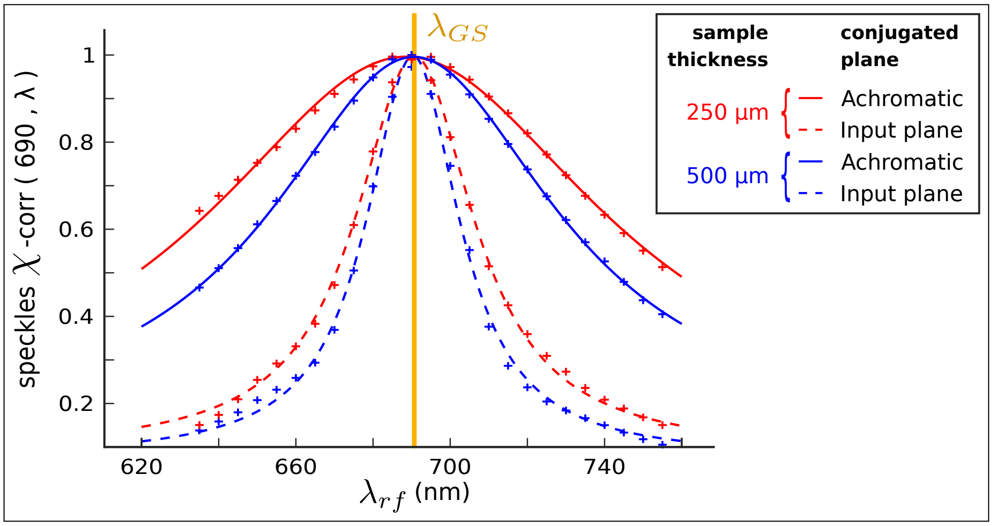

Figure S11: **Spectral cross-correlation product of speckles transmitted through paraffin samples of thicknesses $L = 250\,\mu$m (red) and $L = 500\,\mu$m (blue) when illuminated by a collimated laser beam.** A reference speckle is considered at the arbitrary wavelength $\lambda_{GS} = 690$ nm. Experimental data are fitted using Lorentizan curves ($C(\lambda_{rf}) = 1/[1 + (\lambda_{GS} - \lambda_{rf})^2/w_\lambda^2]$) both in the case when the camera records speckles while conjugated to the virtual entrance plane image (dashed lines) and the virtual achromatic plane image (continuous lines). For $L = 250\,\mu$m (red plots), the measured FWHM are $2w_\lambda(\mathrm{GS}) = 37$ nm and $2w_\lambda(\mathrm{achr}) = 126$ nm in the entrance plane and the achromatic plane, respectively. For $L = 500\,\mu$m (blue plots), the measured FWHM are $2w_\lambda(\mathrm{GS}) = 29$ nm and $2w_\lambda(\mathrm{achr}) = 90$ nm in the entrance plane and the achromatic plane, respectively.

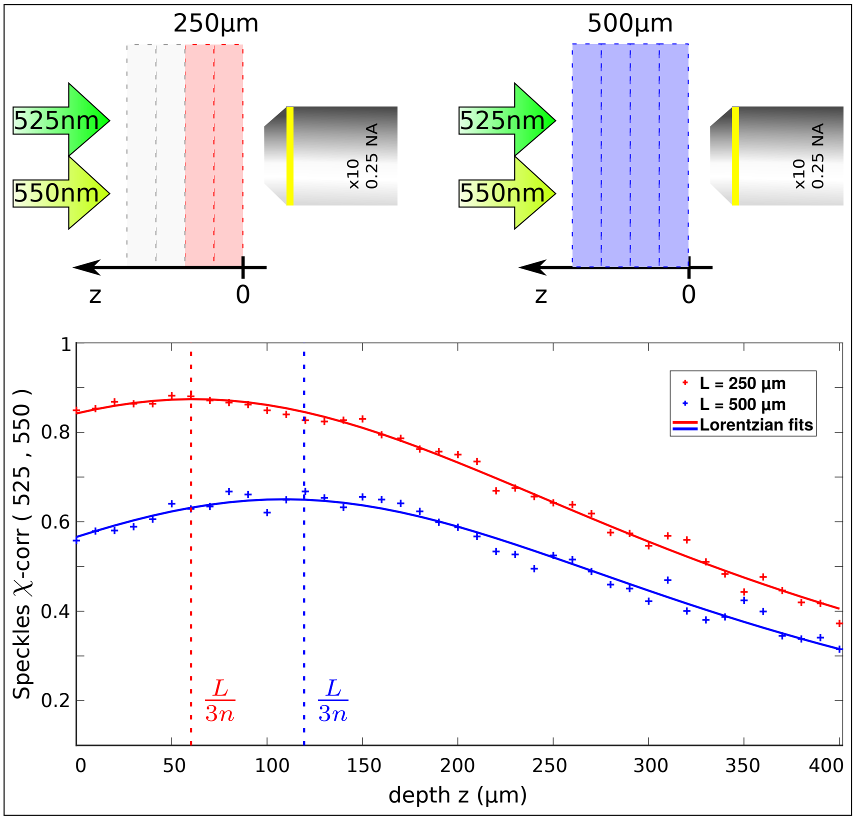

Figure S12: **Achromatic plane location evidenced by axial cross-correlation product of speckles at two nearby wavelengths ($\lambda_1 = 525$ nm and $\lambda_2 = 550$ nm).** The beams are transmitted through paraffin samples of thicknesses $L = 250\ \mu$m (red) and $L = 500\ \mu$m (blue) when illuminated by a collimated laser beam. Experimental data are fitted using Lorentizan curves ($C = 1/[1 + (z - z_{achr})^2/w_z^2]$), both revealing a correlation maximum at $z_{achr} \simeq \frac{L}{3n}$, with L the slab thickness and n the average refractive index of paraffin, in agreement with theory. Noteworthy, the $L/3$ position of the achromatic plane must be corrected by the average refractive index mismatch between the slab (paraffin) and the embedding medium (air). Measured achromatic planes: $z_{achr}(250\ \mu\text{m}) = 61.4\ \mu$m, $z_{achr}(500\ \mu\text{m}) = 109\ \mu$m *vs.* theoretical achromatic planes: $z^{th}_{achr}(250\ \mu\text{m}) = 59.5\ \mu$m, $z^{th}_{achr}(500\ \mu\text{m}) = 119\ \mu$m for an estimated value $n = 1.4$.

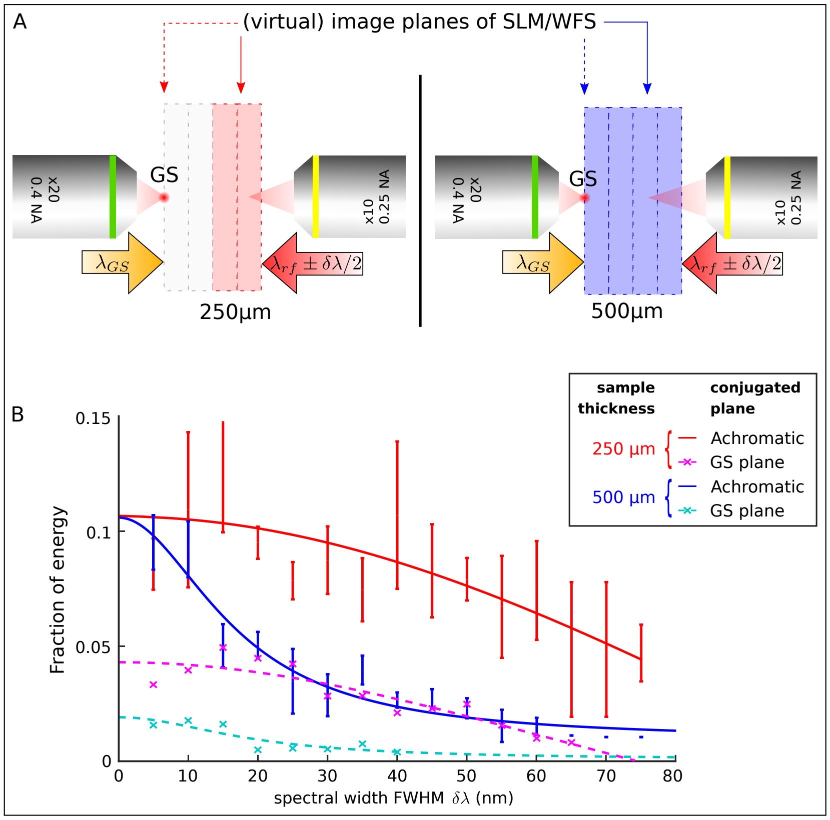

Figure S13: **Effect of the spectral width of the playback laser on the fraction of refocused light energy.** Both for the cases of paraffin samples of thicknesses $L = 250\ \mu m$ (red) and $L = 500\ \mu m$ (blue), the guide-star ($\lambda_{GS} = 690$ nm) was focused at a physical distance of $500\ \mu m$ from the output surface of the sample as shown in (A). DOPC was then achieved using a broadband playback light beam of central wavelength $\lambda_{rf} = \lambda_{GS}$ and of bandwidth $\delta\lambda$ FWHM. Fraction of refocused energy as a function of $\delta\lambda$ is shown in (B) for the SLM/WFS conjugated to the achromatic plane (solid line fits) and the guide-star plane (dashed line fits). Experimental data are fitted using Lorentizan functions $f(\delta\lambda) = \alpha/[1+(\delta\lambda/\Delta\lambda)^2]$, with $\Delta\lambda_{achr}(L = 250) = 70.4$ nm, $\Delta\lambda_{GS}(L = 250) = 44$ nm, $\Delta\lambda_{achr}(L = 500) = 22$ nm, $\Delta\lambda_{GS}(L = 500) = 19.7$ nm. For the measurements in the achromatic plane, a set of three realizations was achieved and the experimental variance is shown as bars.

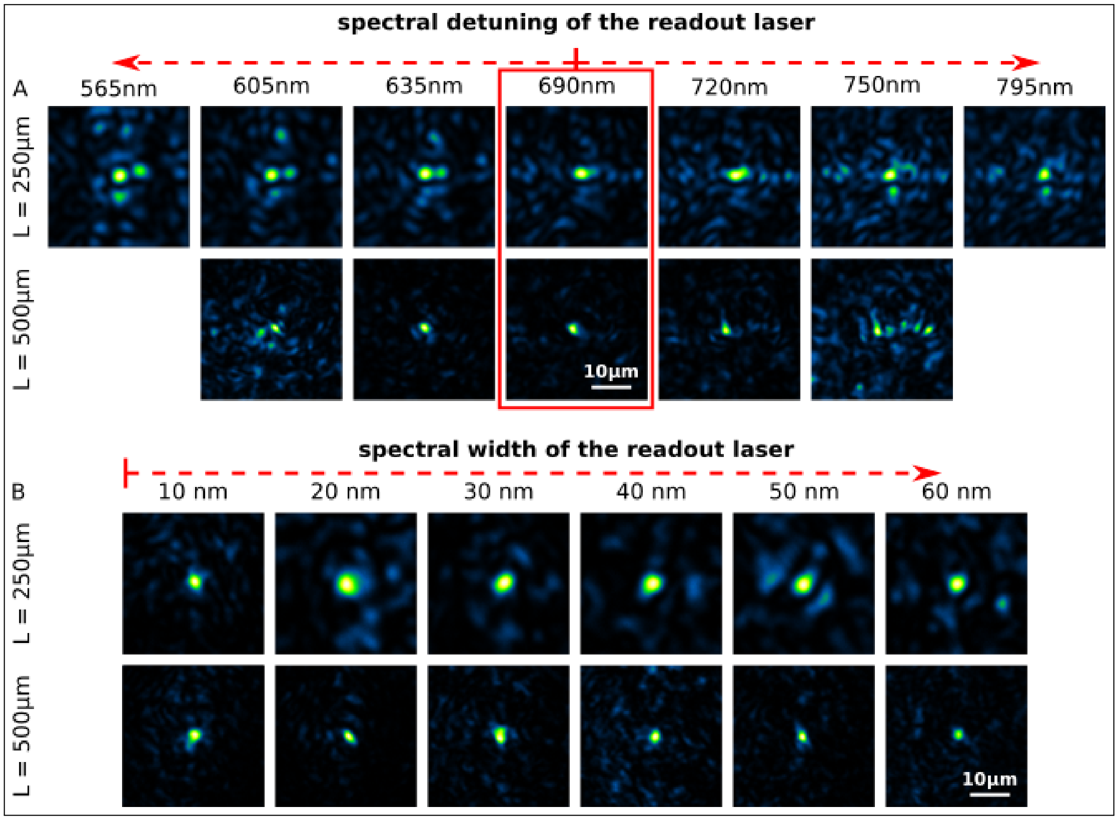

Figure S14: **Images of the focii of the readout laser.** Two paraffin samples of thicknesses $L = 250$ μm and $L = 500$ μm are considered. The playback laser has wavelength detuned from the guide-star laser (A) and spectral width increased (B). In B, the spectrum is centered on the guide-star wavelength. Images in A and B correspond to data-points of curves shown in Fig. 3 and Supp. Fig. S13, respectively.

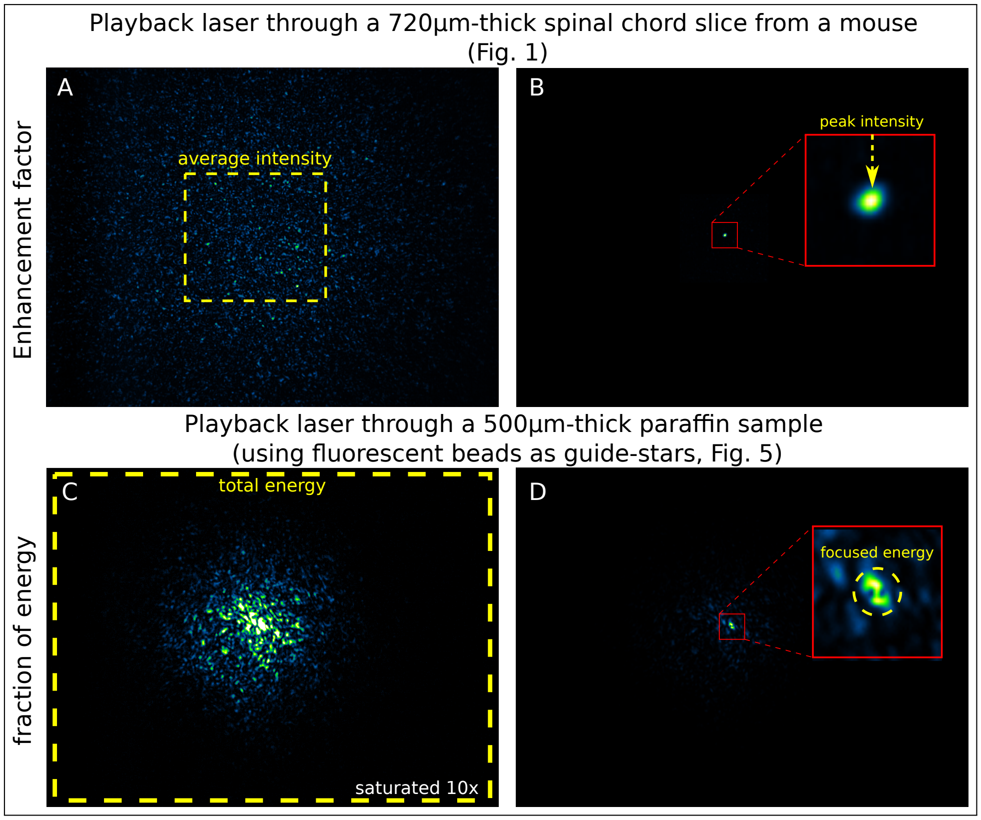

Figure S15: **Experimental estimation of the enhancement factor and the fraction of refocused energy, depending on the scattering sample at play.** For the experiment with the spinal chord slice in Fig. 1, the energy is not fully collected by the objective lens. The enhancement factor is thus considered, defined as the ratio between the peak intensity of the focus (B) and the average speckle intensity (computed in the yellow rectangle where the speckle is uniform in (A)). Conversely, for the DFPC experiment shown in Fig. 5, light is scattered at smaller angles allowing full light-energy collection by the objective lenses and not allowing measuring the peak intensity of the focus because of the focus degradation by the microbead. The fraction of refocused energy can then be measured, and is then computed as the ratio between the energy in a small circle surrounding the bead (D) and the total light energy on the camera (integrating the energy in the yellow rectangle in (C)). In both conditions, the background noise was subtracted.

REFERENCES AND NOTES

1. V. Ntziachristos, Going deeper than microscopy: The optical imaging frontier in biology. *Nat. Methods* **7**, 603–614 (2010).

2. Z. Li, Y. Zheng, X. Diao, R. Li, N. Sun, Y. Xu, X. Li, S. Duan, W. Gong, K. Si, Robust and adjustable dynamic scattering compensation for high-precision deep tissue optogenetics. *Commun. Biol.* **6**, 128 (2023).

3. S. Lal, S. E. Clare, N. J. Halas, Nanoshell-enabled photothermal cancer therapy: Impending clinical impact. *Acc. Chem. Res.* **41**, 1842–1851 (2008).

4. S. L. Jacques, Optical properties of biological tissues: A review. *Phys. Med. Biol.* **58**, R37–R61 (2013).

5. P. Theer, W. Denk, On the fundamental imaging-depth limit in two-photon microscopy. *J. Opt. Soc. Am. A* **23**, 3139–3149, (2006).

6. N. Ji, Adaptive optical fluorescence microscopy. *Nat. Methods* **14**, 374–380 (2017).

7. R. Horstmeyer, H. Ruan, C. Yang, Guidestar-assisted wavefront-shaping methods for focusing light into biological tissue. *Nat. Photonics* **9**, 563–571 (2015).

8. S. M. Popoff, G. Lerosey, R. Carminati, M. Fink, A. C. Boccara, S. Gigan, Measuring the transmission matrix in optics: An approach to the study and control of light propagation in disordered media. *Phys. Rev. Lett.* **104**, 100601 (2010).

9. A. Badon, D. Li, G. Lerosey, A. C. Boccara, M. Fink, A. Aubry, Smart optical coherence tomography for ultra-deep imaging through highly scattering media. *Sci. Adv.* **2**, e1600370 (2016).

10. I. M. Vellekoop, A. Mosk, Focusing coherent light through opaque strongly scattering media. *Opt. Lett.* **32**, 2309–2311 (2007).

11. O. Katz, E. Small, Y. Guan, Y. Silberberg, Noninvasive nonlinear focusing and imaging through strongly scattering turbid layers. *Optica* **1**, 170–174 (2014).

12. M. A. May, N. Barré, K. K. Kummer, M. Kress, M. Ritsch-Marte, A. Jesacher, Fast holographic scattering compensation for deep tissue biological imaging. *Nat. Commun.* **12**, 4340 (2021).

13. M. Cui, C. Yang, Implementation of a digital optical phase conjugation system and its Application to study the robustness of turbidity suppression by phase conjugation. *Opt. Express* **18**, 3444–3455 (2010).

14. B. Blochet, W. Akemann, S. Gigan, L. Bourdieu, Fast wavefront shaping for two-photon brain imaging with large field of view correction. bioRxiv, 2021–09, 2021 [Preprint] (2021). https://doi.org/10.1101/2021.09.06.459064

15. D. Débarre, E. J. Botcherby, T. Watanabe, S. Srinivas, M. J. Booth, T. Wilson, Image-based adaptive optics for two-photon microscopy. *Opt. Lett.* **34**, 2495–2497 (2009).

16. N. Ji, D. E. Milkie, E. Betzig, Adaptive optics via pupil segmentation for high-resolution imaging in biological tissues. *Nat. Methods* **7**, 141–147 (2010).

17. A. Boniface, B. Blochet, J. Dong, S. Gigan, Noninvasive light focusing in scattering media using speckle variance optimization. *Optica* **6**, 1381–1385 (2019).

18. I. M. Vellekoop, E. Van Putten, A. Lagendijk, A. Mosk, Demixing light paths inside disordered metamaterials. *Opt. Express* **16**, 67–80 (2008).

19. B. Rauer, H. B. de Aguiar, L. Bourdieu, S. Gigan, Scattering correcting wavefront shaping for three-photon microscopy. *Opt. Lett.* **47**, 6233–6236 (2022).

20. M. M. Qureshi, J. Brake, H.-J. Jeon, H. Ruan, Y. Liu, A. M. Safi, T. J. Eom, C. Yang, E. Chung, In vivo study of optical speckle decorrelation time across depths in the mouse brain. *Biomed. Opt. Express* **8**, 4855–4864 (2017).

21. Y. Liu, P. Lai, C. Ma, X. Xu, A. A. Grabar, L. V. Wang, Optical focusing deep inside dynamic scattering media with near-infrared time-reversed ultrasonically encoded (true) light. *Nat. Commun.* **6**, 5904 (2015).

22. H. Ruan, M. Jang, C. Yang, Optical focusing inside scattering media with time-reversed ultrasound microbubble encoded light. *Nat. Commun.* **6**, 8968 (2015).

23. D. Aizik, I. Gkioulekas, A. Levin, Fluorescent wavefront shaping using incoherent iterative phase conjugation. *Optica* **9**, 746–754, (2022).

24. Y. Baek, H. B. de Aguiar, S. Gigan, Generalized phase conjugation for incoherent light in complex media. arXiv:2301.13140 [physics.optics] (2023).

25. K. M. Hampson, R. Turcotte, D. T. Miller, K. Kurokawa, J. R. Males, N. Ji, M. J. Booth, Adaptive optics for high-resolution imaging. *Nat. Rev. Methods Primers* **1**, 68 (2021).

26. S. Imperato, F. Harms, A. Hubert, M. Mercier, L. Bourdieu, A. Fragola, Single-shot quantitative aberration and scattering length measurements in mouse brain tissues using an extended-source shack-hartmann wavefront sensor. *Opt. Express* **30**, 15250–15265 (2022).

27. I. M. Vellekoop, M. Cui, C. Yang, Digital optical phase conjugation of fluorescence in turbid tissue. *Appl. Phys. Lett.* **101**, 081108 (2012).

28. D. Akbulut, T. J. Huisman, E. G. van Putten, W. L. Vos, A. P. Mosk, Focusing light through random photonic media by binary amplitude modulation. *Opt. Express* **19**, 4017–4029 (2011).

29. Y. Liu, C. Ma, Y. Shen, J. Shi, L. V. Wang, Focusing light inside dynamic scattering media with millisecond digital optical phase conjugation. *Optica* **4**, 280–288 (2017).

30. M. Rueckel, J. A. Mack-Bucher, W. Denk, Adaptive wavefront correction in two-photon microscopy using coherence-gated wavefront sensing. *Proc. Natl. Acad. Sci. U.S.A.* **103**, 17137–17142 (2006).

31. X. Tao, B. Fernandez, O. Azucena, M. Fu, D. Garcia, Y. Zuo, D. C. Chen, J. Kubby, Adaptive optics confocal microscopy using direct wavefront sensing. *Opt. Lett.* **36**, 1062–1064 (2011).

32. P. Bon, J. Linarès-Loyez, M. Feyeux, K. Alessandri, B. Lounis, P. Nassoy, L. Cognet, Self-interference 3d super-resolution microscopy for deep tissue investigations. *Nat. Methods* **15**, 449–454 (2018).

33. J. Primot, L. Sogno, Achromatic three-wave (or more) lateral shearing interferometer. *J. Opt. Soc. Am. A* **12**, 2679–2685 (1995).

34. P. Bon, G. Maucort, B. Wattellier, S. Monneret, Quadriwave lateral shearing interferometry for quantitative phase microscopy of living cells. *Opt. Express* **17**, 13080–13094 (2009).

35. G. A. Tyler, Reconstruction and assessment of the least-squares and slope discrepancy components of the phase. *J. Opt. Soc. Am. A* **17**, 1828–1839 (2000).

36. N. B. Baranova, B. Y. Zel’Dovich, A. V. Mamaev, N. F. Pilipetskiĭ, V. V. Shkukov, Dislocations of the wavefront of a speckle-inhomogeneous field (theory and experiment). *ZhETF Pisma Redaktsiiu* **33**, 206 (1981).

37. J. F. Nye, M. V. Berry, F. C. Frank, Dislocations in wave trains. *Proc. R. Soc. Lond. A Math. Phys. Sci.* **336**, 165–190 (1974).

38. M. Pascucci, G. Tessier, V. Emiliani, M. Guillon, Superresolution imaging of optical vortices in a speckle pattern. *Phys. Rev. Lett.* **116**, 093904 (2016).

39. D. L. Fried, Branch point problem in adaptive optics. *J. Opt. Soc. Am. A* **15**, 2759–2768, (1998).

40. L. Huang, M. Idir, C. Zuo, K. Kaznatcheev, L. Zhou, A. Asundi, Comparison of two-dimensional integration methods for shape reconstruction from gradient data. *Opt. Lasers Eng.* **64**, 1–11 (2015).

41. W. J. Wild, E. O. L. Bigot, Rapid and robust detection of branch points from wave-front gradients. *Opt. Lett.* **24**, 190–192 (1999).

42. K. Murphy, C. Dainty, Comparison of optical vortex detection methods for use with a shack-hartmann wavefront sensor. *Opt. Express* **20**, 4988–5002 (2012).

43. É. O. Le Bigot, W. J. Wild, E. J. Kibblewhite, Reconstruction of discontinuous light-phase functions. *Opt. Lett.* **23**, 10–12 (1998).

44. F. A. Starikov, G. G. Kochemasov, S. M. Kulikov, A. N. Manachinsky, N. V. Maslov, A. V. Ogorodnikov, S. A. Sukharev, V. P. Aksenov, I. V. Izmailov, F. Y. Kanev, V. V. Atuchin, I. S. Soldatenkov, Wavefront reconstruction of an optical vortex by a hartmann-shack sensor. *Opt. Lett.* **32**, 2291–2293 (2007).

45. K. Murphy, D. Burke, N. Devaney, C. Dainty, Experimental detection of optical vortices with a shack-hartmann wavefront sensor. *Opt. Express* **18**, 15448–15460 (2010).

46. T. Wu, P. Berto, M. Guillon, Reference-less complex wavefields characterization with a high-resolution wavefront sensor. *Appl. Phys. Lett.* **118**, 251102 (2021).

47. S. Schott, J. Bertolotti, J.-F. Léger, L. Bourdieu, S. Gigan, Characterization of the angular memory effect of scattered light in biological tissues. *Opt. Express* **23**, 13505–13516 (2015).

48. X. Cheng, Y. Li, J. Mertz, S. Sakadžić, A. Devor, D. A. Boas, L. Tian, Development of a beam propagation method to simulate the point spread function degradation in scattering media. *Opt. Lett.* **44**, 4989–4992 (2019).

49. P. Arjmand, O. Katz, S. Gigan, M. Guillon, Three-dimensional broadband light beam manipulation in forward scattering samples. *Opt. Express* **29**, 6563–6581 (2021).

50. A. G. Vesga, M. Hofer, N. K. Balla, H. B. De Aguiar, M. Guillon, S. Brasselet, Focusing large spectral bandwidths through scattering media. *Opt. Express* **27**, 28384–28394 (2019).

51. L. Zhu, J. B. de Monvel, P. Berto, S. Brasselet, S. Gigan, M. Guillon, Chromato-axial memory effect through a forward-scattering slab. *Optica* **7**, 338–345 (2020).

52. I. N. Papadopoulos, J.-S. Jouhanneau, J. F. Poulet, B. Judkewitz, Scattering compensation by focus scanning holographic aberration probing (f-sharp). *Nat. Photonics* **11**, 116–123 (2017).

53. A. Badon, V. Barolle, K. Irsch, A. C. Boccara, M. Fink, A. Aubry, Distortion matrix concept for deep optical imaging in scattering media. *Sci. Adv.* **6**, eaay7170 (2020).

54. B. Mastiani, G. Osnabrugge, I. M. Vellekoop, Wavefront shaping for forward scattering. *Opt. Express* **30**, 37436–37445 (2022).

55. A. Thendiyammal, G. Osnabrugge, T. Knop, I. M. Vellekoop, Model-based wavefront shaping microscopy. *Opt. Lett.* **45**, 5101–5104 (2020).

56. J. Li, D. R. Beaulieu, H. Paudel, R. Barankov, T. G. Bifano, J. Mertz, Conjugate adaptive optics in widefield microscopy with an extended-source wavefront sensor. *Optica* **2**, 682–688 (2015).

57. C. K. Mididoddi, R. A. Lennon, S. Li, D. B. Phillips, High-fidelity off-axis digital optical phase conjugation with transmission matrix assisted calibration. *Opt. Express* **28**, 34692–34705 (2020).

58. G. Osnabrugge, R. Horstmeyer, I. N. Papadopoulos, B. Judkewitz, I. M. Vellekoop, Generalized optical memory effect. *Optica* **4**, 886–892 (2017).

59. M. Jang, C. Yang, I. Vellekoop, Optical phase conjugation with less than a photon per degree of freedom. *Phys. Rev. Lett.* **118**, 093902 (2017).

60. P. Bon, S. Monneret, B. Wattellier, Noniterative boundary-artifact-free wavefront reconstruction from its derivatives. *Appl. Optics* **51**, 5698–5704 (2012).

61. I. Freund, '1001' correlations in random wave fields. *Waves Random Complex Media* **8**, 119–158 (1998).

62. C. Dunsby, P. M. W. French, Techniques for depth-resolved imaging through turbid media including coherence-gated imaging. *J. Phys. D Appl. Phys.* **36**, R207–R227 (2003).

63. J.-H. Park, W. Sun, M. Cui, High-resolution in vivo imaging of mouse brain through the intact skull. *Proc. Natl. Acad. Sci. U.S.A.* **112**, 9236–9241 (2015).

64. M. Dennis, K. O'Holleran, M. Padgett, Chapter 5 singular optics: Optical vortices and polarization singularities. *Prog. Opt.* **53**, 293–363 (2009).

65. M. Xu, R. R. Alfano, Random walk of polarized light in turbid media. *Phys. Rev. Lett.* **95**, 213901 (2005).

66. G. Baffou, Quantitative phase microscopy using quadriwave lateral shearing interferometry (qlsi): Principle, terminology, algorithm and grating shadow description. *J. Phys. D Appl. Phys.* **54**, 294002 (2021).

67. S. Yang, E. Papagiakoumou, M. Guillon, V. de Sars, C.-M. Tang, V. Emiliani, Three-dimensional holographic photostimulation of the dendritic arbor. *J. Neural Eng.* **8**, 046002 (2011).
